# Predicting need for escalation of care or death from repeated daily clinical observations and laboratory results in patients with SARS-CoV-2

**DOI:** 10.1093/aje/kwac126

**Published:** 2022-07-22

**Authors:** Colin J Crooks, Joe West, Andrew Fogarty, Joanne R Morling, Matthew J Grainge, Sherif Gonem, Mark Simmonds, Andrea Race, Irene Juurlink, Steve Briggs, Simon Cruickshank, Susan Hammond-Pears, Timothy R Card

**Affiliations:** Translational Medical Sciences, School of Medicine, University of Nottingham, NG7 2UH; NIHR Nottingham Biomedical Research Centre (BRC), Nottingham University Hospitals NHS Trust and the University of Nottingham, NG7 2UH; Nottingham University Hospitals NHS Trust, NG7 2UH; Lifespan and Population Health Sciences, School of Medicine, University of Nottingham, NG5 1PB; NIHR Nottingham Biomedical Research Centre (BRC), Nottingham University Hospitals NHS Trust and the University of Nottingham, NG7 2UH; Nottingham University Hospitals NHS Trust, NG7 2UH; East Midlands Academic Health Science Network, University of Nottingham, Nottingham, NG7 2TU; Lifespan and Population Health Sciences, School of Medicine, University of Nottingham, NG5 1PB; NIHR Nottingham Biomedical Research Centre (BRC), Nottingham University Hospitals NHS Trust and the University of Nottingham, NG7 2UH; Nottingham University Hospitals NHS Trust, NG7 2UH; Lifespan and Population Health Sciences, School of Medicine, University of Nottingham, NG5 1PB; NIHR Nottingham Biomedical Research Centre (BRC), Nottingham University Hospitals NHS Trust and the University of Nottingham, NG7 2UH; Nottingham University Hospitals NHS Trust, NG7 2UH; Translational Medical Sciences, School of Medicine, University of Nottingham, NG7 2UH; Nottingham University Hospitals NHS Trust, NG7 2UH; Division of Respiratory Medicine, School of Medicine, University of Nottingham, NG5 1PB; Nottingham University Hospitals NHS Trust, NG7 2UH; Nottingham University Hospitals NHS Trust, NG7 2UH; Nottingham University Hospitals NHS Trust, NG7 2UH; Nottingham University Hospitals NHS Trust, NG7 2UH; Nottingham University Hospitals NHS Trust, NG7 2UH; Nottingham University Hospitals NHS Trust, NG7 2UH; East Midlands Academic Health Science Network, University of Nottingham, Nottingham, NG7 2TU; Lifespan and Population Health Sciences, School of Medicine, University of Nottingham, NG5 1PB; NIHR Nottingham Biomedical Research Centre (BRC), Nottingham University Hospitals NHS Trust and the University of Nottingham, NG7 2UH; Nottingham University Hospitals NHS Trust, NG7 2UH

**Keywords:** Critical Care, Survival Analysis, SARS-CoV-2, COVID-19, Mortality

## Abstract

We compared the performance of prognostic tools for SARS-CoV-2 using parameters fitted either at time of admission or across all time points of an admission. This cohort study used clinical data to model the dynamic change in prognosis of SARS-CoV-2 in a single hospital in England including all patients admitted from 1st February 2020 until 31^st^ December 2020, and then followed up for ICU admission, death, or discharge from hospital for 60 days. We incorporated clinical observations and blood tests into two-time varying Cox proportional hazards models predicting daily 24–48-hour risk of admission to ICU for those eligible, or death for those ineligible for escalation. To develop the model 491 patients were eligible for ICU escalation and 769 were ineligible for escalation. Our model had good discrimination of daily risk of ICU admission in the validation cohort (n = 1141, C statistic = 0.91 (95% CI 0.89 -0.94)) and performed better than other scores (NEWS2, ISCARIC 4C) calculated using only parameters on admission, but overestimated escalation (calibration slope 0.7). A bespoke daily SARS-CoV-2 escalation risk prediction score can predict need for clinical escalation better than a generic early warning score or a single estimation of risk calculated at admission.

## Introduction

The Severe Acute Respiratory Syndrome CoronaVirus 2 (SARS-CoV-2) pandemic has brought some health systems to a state of near collapse [1] and has increased the risk of death from other diseases due to the diversion of resources[2,3].

During the first wave of the pandemic, in 2020, many prognostic scores[4–6] like the International Severe Acute Respiratory Infection Coronavirus Clinical Characterisation Consortium mortality score (ISARIC 4C)[5,7] were created bespoke for SARS-CoV-2 but were based on information from a single time point (admission). However, clinicians make clinical decisions regarding escalation of care throughout the disease course. Other scores that were used aimed at more dynamic use throughout the disease course, but were not disease specific (such as National Early Warning Score (NEWS2)[8]). A number of these scores perform reasonably, with the area under the receiver operating characteristic curve of 0.77 (0.76-0.77) for the validation cohort of ISARIC 4C mortality[5], 0.77 (0.76-0.78) for the validation cohort of ISARIC 4C deterioration[7], and for NEWS-2 the Area Under the Receiver Operating Characteristic curve (AUC) varied between hospitals from 0.623 to 0.815[9].

A score that is both dynamic (i.e. calculated daily using all available clinical measurements) and optimised for SARS-CoV-2 might therefore perform better than the alternatives and be of greater value to both clinicians and hospital managers. We therefore aimed to derive and validate a

ORIGINAL UNEDITED MANUSCRIPT

disease severity score based on daily clinical observations and blood measurements which would predict next day Intensive Care Unit (ICU) or mortality for those eligible for escalation, and next day mortality for those ineligible for escalation. We also planned to compare the performance characteristics of this new score to the NEWS2 and ISARIC 4C scores.

Methods

We carried out and reported this study in accordance with the Transparent Reporting of a multivariable prediction model for Individual Prognosis or Diagnosis (TRIPOD) guidelines[10].

Study design, setting and populations

This retrospective, observational cohort study was conducted at Nottingham University Hospitals (NUH) National Health Service (NHS) Trust, United Kingdom (UK). All admitted patients were identified with a confirmed SARS-CoV-2 diagnosis via either; i) a positive result on polymerase chain reaction (PCR) testing of a nasopharyngeal sample, or ii) a recorded clinical diagnosis based on typical radiological features of SARS-CoV-2. Patients were included from 21 February 2020 (the date of disease onset of the first known case at NUH) until 30 June 2020 inclusive for the derivation cohort, and then until 31st December 2020 as the validation cohort. All follow up continued until the earliest of discharge from hospital, or day prior to admission to ICU or death until 28 February 2021. We split this cohort to derive two separate models based on the attending physicians' decision on whether patients were eligible or ineligible for escalation to ICU. This decision was made as part of a patient's routine clinical care based on their frailty and co-morbidity. All demographic information, comorbidities, ceiling of care decisions, laboratory tests, and clinical observations were extracted for the identified hospital admissions. Patients entered the derivation cohort for the prediction models at the earliest time from when both

ORIGINAL UNEDITED MANUSCRIPT

clinical observations and blood tests (blood count, urea, and electrolytes) were available after SARS-CoV-2 diagnosis.

Statistical Analysis

*Outcome*

We defined two cohorts: first for those patients eligible for escalation of respiratory support in ICU, the combined outcome was defined of either first ICU admission or death within 60 days from first date of SARS-CoV-2. For those patients ineligible for escalation of respiratory support in ICU, death alone was defined as an outcome.

*Baseline covariates*

Age was categorised as a linear variable, a quadratic transformation and 20-year categories (20-39, 40-59, 60-79, >79 years), and likelihood ratio tests used to select the best fit. The presence of co-morbidity was categorised by the recording of any co-morbidity in the Charlson index[11].

*Time varying covariates*

We aimed to investigate whether time varying measures allowed the model to better capture the dynamic changes of risk in comparison to admission only scores, like ISARIC-4C, or scores based on a snapshot of point estimates, like NEWS2. Therefore, daily summary measures of blood tests and observations were derived as follows; i) The daily mean of each blood test and the worst daily value for clinical observations to capture the current magnitude of each measure, ii) the daily change (difference between first and last measurement within a day) to capture the short term within day trend of each measure, and iii) the lagged change in the mean or worst value from the previous day to capture the longer term between day trend. Last observed measurements were carried forward for calculating the daily summary measures, and patient days prior to measurements being available were excluded. These lagged daily summary

ORIGINAL UNEDITED MANUSCRIPT

measures were then used to predict outcomes on the following day. On the first day of admission when lagged measures were not calculable, the daily measures from that same day of admission were used.

To assess the effect of excluding patient days prior to measurements being available, we performed a sensitivity analysis imputing missing data on day of admission to derive thirty imputation data sets with multi-level multiple imputations by chained equations with the R package ‘mice’ [12].

*Model selection and assumptions*

Covariates for a time varying Cox proportional hazards model were selected using both forward and backward steps with Akaike information criterion (AIC) as a measure of goodness of fit (using the R packages survival[13] and MASS[14]), and bootstrapping the process 100 times to assess optimism and the consistency with which parameters were selected. The proportional hazards assumption was assessed by visually examining the Schoenfeld residuals and testing the covariate for a fitted slope versus time. For the missing data sensitivity analysis the models were refitted to the imputed datasets and pooled using Rubin’s rules.

*Sample size*

Prior to the study, the outcome prevalence was anticipated to be 0.12-0.27 and a lower bound for the new model's acceptable R-squared value as 0.15. This estimated a sample size of around 500 patients using 10 candidate predictors as shown in Web Table 1.

*Internal validation and comparison to NEWS2 and ISARIC4C*

The performance of the model was tested in the development of the score using the C statistic fitted with leave one out cross validation in the derivation cohort across different time points.

ORIGINAL UNEDITED MANUSCRIPT

This was performed by sequentially excluding each patient in turn with all their observations. We then validated the calibration and performance of the score in the 2nd wave validation cohort using both the C statistic and integrated Brier score (an averaged measure between 0 to 1 of the difference between the observed and predicted survival adjusted for censoring and time varying covariates [15]). Finally, we compared our score with the performance of NEWS2 and ISARIC 4C implemented using the published methods. A further sensitivity analysis was undertaken validating with only those patients with confirmatory PCR for SARS-CoV-2.

All analyses were performed using version 4.0.3 of the R programming language (R project for Statistical Computing; R Foundation, Indianapolis, Indiana). Approval for this work was granted via an NUH Clinical Effectiveness Team audit (reference: 20-153C), the NUH Caldicott Guardian, Data Protection Impact Assessment (reference: 436) and as a research study (ethics approval) via the NHS Health Research Authority (HRA) Integrated Research Application System (IRAS) (reference: 282490).

Results

The combined derivation and validation cohort demographic, baseline characteristics and mortality outcomes are shown in table 1. Overall, 3,898 patients were admitted and the key differences apparent between the 1$^{st}$ and 2$^{nd}$ wave cohorts were that in the 2$^{nd}$ wave the median age was slightly lower (1$^{st}$ wave: 76 versus 2$^{nd}$ wave: 72) and 30-day mortality was substantially lower (1$^{st}$ wave: 25% versus 2$^{nd}$ wave: 20%).

First wave derivation cohort

From 21 February 2020 until 30 June 2020 1,443 patients were admitted to Nottingham University Hospitals with clinically confirmed SARS-CoV-2. The daily status of these patients is

ORIGINAL UNEDITED MANUSCRIPT

shown in Web Figure 1 by day of disease course (measured from the day SARS-CoV-2 was first recorded).

Of those patients in the derivation cohort 1,040 (72%) had a confirmatory PCR with the remainder having a clinical diagnosis from typical radiological features (Web Table 2). 491 patients were eligible for escalation of respiratory support with both blood tests and observations recorded during their admission in the time before any escalation to ICU or death (Web Figure 2a). 90 of these patients were escalated or died whilst an inpatient during 60 days of first diagnosis date for the derivation of the eligible for escalation to ICU model. For derivation of the ineligible for escalation to ICU model 769 patients had observations and blood tests available after earliest diagnosis SARS-CoV-2 date (Web Figure 2a).

Second wave validation cohort

From 1st July 2020 until 31st December 2020, 2,455 patients were admitted with a clinical SARS-CoV-2 diagnosis (2,048 with PCR positive tests), of whom 1,356 were eligible for escalation to ICU and 1,032 ineligible for escalation to ICU (Web Figure 2b).

Patients eligible for escalation in 1$^{st}$ wave: Predicting daily risk of next day ICU admission or death

Table 2 shows initial measurements and missing data for patients at the earliest time point after diagnosis of SARS-CoV-2 when both blood and clinical observations were available. Web Figure 3 shows how selected observations and blood results then varied during the admission stratified by patients' final outcomes.

Modelling daily summary measures of full blood count, urea and electrolytes, and observations as described in the methods showed a model with a quadratic and linear term for age was a

ORIGINAL UNEDITED MANUSCRIPT

statistically better fit than categorical (likelihood ratio test p = 0.03). Blood cell counts were transformed to the log scale due to positive skew.

The final selected model predicting next day escalation or death (table 3) had an overall concordance of 0.91 (95% Confidence Interval (CI) 0.87-0.94). The adjustment for optimism using the bootstrapped uniform shrinkage factor was estimated at 0.70 (Interquartile range (IQR) 0.62-0.82). The sensitivity analysis imputing missing data attenuated but did not substantially alter the covariates (table 3). The integrated Brier score confirmed a low mean squared error of 0.01. Residual plots testing the proportional hazards association are shown in the Web Figure 4 and Web Table 3.

Concordance did not alter with cross validation using bootstrapped samples (0.90 (IQR 0.88-0.91) and remained high across the follow up time (Web Figure 5). The corresponding discrimination was lower for both the ISARIC 4C mortality score C statistic = 0.64 (0.58-0.69) and for the NEWS2 score C statistic = 0.86 (0.82-0.90). Restricting the population to just those that had a SARS-CoV-2 PCR positive test did not alter the discrimination (0.89 (IQR 0.87-0.91)). The final algorithm is shown in the Web Table 4.

Patients ineligible for escalation in 1st wave: Predicting next day mortality

For patients not eligible for escalation to ICU a separate model was built predicting only next day mortality (table 3). The model’s discrimination in the derivation cohort with leave one out cross validation using bootstrapped samples was 0.86 (0.84 - 0.89) and remained high throughout follow up (Web Figure 6). Baseline survival plots are shown in Web Figure 7. The final algorithm is shown in the Web Table 3. The integrated Brier score confirmed a low mean

ORIGINAL UNEDITED MANUSCRIPT

squared error of 0.04. Residual plots testing the proportional hazards association are shown in the Web Figure 8 and Web Table 5.

Calibration and Comparison with existing scores in first wave

The magnitude of the two derived scores tracked the observed outcomes for inpatients eligible for ICU (figure 1) and ineligible for ICU (figure 2) in the derivation first wave cohort.

Second Wave Validation

For patients eligible for escalation to ICU in the second wave validation cohort, discrimination remained high with a concordance of 0.91 (95% CI 0.89 -0.94) (Web Figure 9a) and to a less extent for ISARIC-4C mortality (C statistic = 0.70 (95% CI 0.66-0.75)) and NEWS2 (C statistic = 0.89 (95% CI 0.86 – 0.92). The integrated Brier score confirmed the mean squared error remained low at 0.01.

To assess calibration, Web Figure 9 shows the derived score overestimated next day escalation in the second wave validation cohort with a calibration slope of 0.68. In Web Table 6 the negative predictive value remained above 98% for all levels of the derived score, and the positive predictive value was over 40% when the linear predictor was above 4.

For patients ineligible for escalation to ICU the model predicting next day mortality had a discrimination of 0.88 (95% CI 0.86-0.89) and calibration slope of 0.69 (Web Figure 9b). In comparison, the discrimination of the daily NEWS score for next day mortality was 0.81 (95% CI 0.78-0.83), and the ISARIC-4C score 0.79 (95% CI 0.76-0.81). In Web Table 6 the negative predictive value remained above 97% for all levels of the derived score, and the positive predictive value was 60% when the linear predictor was above 4. The integrated Brier score showed the mean squared error was higher at 0.05.

ORIGINAL UNEDITED MANUSCRIPT

The magnitude of the two derived scores tracked the observed outcomes for inpatients eligible for ICU (figure 3) and ineligible for ICU (figure 4) in the second wave validation cohort, but at lower thresholds than the derivation cohort reflecting the change in calibration.

## Discussion

### Main findings

This study incorporated daily clinical and laboratory measurements with baseline characteristics to predict the daily dynamic risk of next day escalation of care or mortality in patients with SARS-CoV-2 with better precision throughout the hospital stay than using the same parameters from a model derived only at admission. The validation showed excellent discrimination and accuracy (as measured by the integrated Brier score) but it over predicted death and escalation at the thresholds taken from the derivation cohort. This is likely to reflect the change in demographics and clinical practice between the first and second UK wave, given changes in escalation practice[16,17] and the introduction of the use of steroids[18,19]. Our results suggest that using a dynamic score derived from daily blood and clinical measurements could provide better prediction of the need for escalation of care in SARS-CoV-2 than scores derived from similar parameters measured at a single time point i.e. on admission.

### Strengths and weaknesses

Our study included all patients who were admitted to a large teaching hospital in the Midlands of the UK serving a population that covers metropolitan, suburban and rural areas throughout an eight-month period of 2020. The richness and uniformity of our data is a strength of a single centre, but it is gained at the cost of limiting our analyses to one organisation and therefore the decisions of one cohort of clinicians. This leads to questions regarding generalisability which can

ORIGINAL UNEDITED MANUSCRIPT

only be answered by external validation. However, the diverse population of Nottingham as a representative cross-section of the UK population and the standardisation of care across the NHS suggest that our findings will be replicable.

Through our use of electronic patient record systems, we had access to comprehensive sociodemographic, clinical and laboratory variables including all measurements recorded electronically through the patient's admission. We also had available complete follow up for escalation of care, death (including out of hospital death) and discharge from hospital for 60 days from admission and importantly, therefore, have little bias due to missing outcomes, loss to follow up, or other common biases of observational cohorts.

The missing exposure data that was observed in the cohorts reflects clinical decision making, for example patients who were frail so had compassionate care without imposing blood tests and observations, patients who were too well to keep in hospital for blood tests and observations, and patients who were escalated on admission so did not have measurements available in the pre-event observation time. Therefore, the missing data was not at random, and this is demonstrated by the attenuation of some of the associations in the multiple imputation sensitivity analysis. For our implementation locally we only used the model derived from patients with clinical observations and blood tests available in the pre-escalation period, as this had the most clinical relevance for patients being actively managed with clinical equipoise in their care.

Following the development and validation of our score in 2020, there have been many developments in the management of SARS-CoV-2 including; new treatments (for which we do not have electronic recording), vaccinations (which began after our study validation cohort at the

ORIGINAL UNEDITED MANUSCRIPT

end of 2021), and SARS-CoV-2 variants (which did not reach significant levels in the UK during the 2020 study period).Trigger thresholds for severe disease from our score should therefore be monitored and updated locally depending on the patient population and setting as shown in our calibration results. However, throughout 2021 our score as presented in this paper, has continued to correctly discriminate between those patients who have more severe disease and those who have less severe disease. Ongoing audit of the score's implementation within our hospital trust for the first 10 months of 2021 (to allow complete 60 day follow up) showed discrimination remained high (C = 0.92, as measured by the C statistic for next day ICU admission), compared to NEWS (C = 0.86) and ISARIC 4C (C = 0.67). This demonstrated clinical markers of severity remained the same for patients who become sick, whilst changes in vaccination, variants and treatment might reduce the number of people reaching those markers of severity.

Interpretation

Our report is best compared to other large population-based studies from single cities or regions around the world that have reported their experience through the SARS-CoV-2 pandemic[20–27] and the relevant UK studies[28–30]. The distribution of sociodemographic risk factors and their association with poor prognosis with respect to age and sex are similar to these studies. Our risk prediction model is unique in using longitudinal daily clinical and laboratory measures to estimate the next day need for escalation of care or death. In that respect, we cannot compare it directly to other published risk models, but in relation to those derived within UK populations it performs better[5,7,9,30] and for reasons stated above is at low risk of bias. In particular, compared to the robustly developed ISCARIC 4C mortality prediction score[5] and the ISARIC 4C deterioration score[7] our model performs better on a daily basis – showing the value of incorporating repeated measurements of clinical observations and blood results into the

ORIGINAL UNEDITED MANUSCRIPT

prediction of prognosis for patients admitted to hospital with SARS-CoV-2. We currently use these models integrated into the data warehouse within our hospital to provide a live dynamic overview of the COVID-19 inpatient cohort by current severity (as opposed to admission severity identified by other scores), and to identify locations within the hospital with higher burdens of severe COVID-19.

## Conclusions

We have shown that incorporating daily measurements of clinical observations and blood tests improves the accuracy of the prediction of prognosis in secondary care patients with SARS-CoV-2 compared to similar scoring systems that are based on the use of data from only a single point in time.

Table 1. Sociodemographic and other characteristics on admission of the derivation cohort with earliest date of confirmed SARS-CoV2 diagnosis between 21 February 2020 until 30 June 2020, and second wave validation cohort with earliest date of confirmed SARS-CoV2 diagnosis before 31 December 2020 (followed up until 31 January 2021)

| Cohort characteristic | Admission before 30th June 2020 (Derivation, No. = 1443) | | Admission after 1st July 2020 (Validation, No. = 2455) | |
|---|---|---|---|---|
| | No. | % | No. | % |
| Age (years) [a] | 76 (61, 85) | | 72 (54, 83) | |
| Male | 751 | 52 | 1,255 | 51 |
| Other or not stated ethnic group | 255 | 18 | 491 | 20 |
| Black/Mixed ethnic group | 55 | 4 | 77 | 3 |
| Indian/Pakistani ethnic group | 56 | 4 | 144 | 6 |
| White ethnic group | 1,077 | 75 | 1,743 | 71 |
| 30 day mortality | 365 | 25 | 498 | 20 |
| Died out of hospital | 41 | 3 | 42 | 2 |
| 30 day ICU admission | 151 | 10 | 258 | 11 |
| Length of stay, days [a] | 8 (3, 16) | | 9 (3, 20) | |
| For escalation/CPR | 620 | 43 | 1,422 | 58 |
| NEWS2 [a] | 3 (2, 5) | | 3 (1, 4) | |
| ISARIC-4C [a] | 10 (7, 12) | | 9 (5, 11) | |
| BMI <20 | 259 | 18 | 393 | 16 |
| BMI >30 | 382 | 26 | 671 | 27 |
| Smoking | 160 | 11 | 313 | 13 |
| Vaping | 67 | 5 | 147 | 6 |
| Hazardous alcohol risk | 202 | 14 | 358 | 15 |
| Charlson Index [a] | 2 (1, 3) | | 1 (0, 3) | |

Abbreviations: Severe Acute Respiratory Syndrome CoronaVirus 2 (SARS-CoV2), Number (No.), Intensive Care Unit (ICU), Body Mass Index (BMI)

[a]Values are expressed as median (interquartile range)

ORIGINAL UNEDITED MANUSCRIPT

Table 2. Initial blood test results and observations of the 1391 patients with confirmed SARS-COV-2 and inpatient time observed prior to any escalation to ICU, stratified by eligibility for escalation of care to ICU and worst outcome within 60 days of diagnosis, 21 February 2020 until 30 June 2020 (derivation cohort). Blood tests and observations after ICU admission were excluded. The interquartile range of the time from first confirmed SARS-Cov-2 to the time when clinical observations, blood count and electrolyte blood tests were all available was 0 – 25 hours.

| Outcome by 60 days: | Not for escalation | | | | | | Eligible for escalation | | | | | | | | |
|---|---|---|---|---|---|---|---|---|---|---|---|---|---|---|---|
| | Survived (No. = 461) | | | Died (No. = 345) | | | Not escalated (No. = 440) | | | ICU admission (No. = 70) | | | Died (No. = 75) | | |
| Blood test or clinical observation[a]: | Median (IQR) | Not measured | | Median (IQR) | Not measured | | Median (IQR) | Not measured | | Median (IQR) | Not measured | | Median (IQR) | Not measured | |
| | | No. | % | | No. | % | | No. | % | | No. | % | | No. | % |
| Haemoglobin (g/L) | 120 (106, 133) | 7 | 2 | 117 (102, 136) | 10 | 3 | 128 (114, 143) | 34 | 8 | 133 (114, 145) | 13 | 19 | 120 (104, 136) | 8 | 11 |
| Platelets ($10^9$/L) | 236 (177, 295) | 7 | 2 | 214 (162, 295) | 10 | 3 | 233 (176, 311) | 34 | 8 | 227 (182, 333) | 16 | 23 | 226 (158, 327) | 9 | 12 |
| Neutrophils ($10^9$/L) | 6.1 (4.0, 9.1) | 7 | 2 | 6.7 (4.7, 9.9) | 10 | 3 | 5.1 (3.4, 7.8) | 34 | 8 | 6.4 (4.6, 9.1) | 13 | 19 | 6.8 (4.5, 10.9) | 8 | 11 |
| Lymphocytes ($10^9$/L) | 0.9 (0.6, 1.4) | 7 | 2 | 0.8 (0.5, 1.1) | 10 | 3 | 1.1 (0.8, 1.6) | 34 | 8 | 0.9 (0.7, 1.1) | 16 | 23 | 0.9 (0.6, 1.3) | 9 | 12 |
| Sodium (mmol/L) | 136 (133, 139) | 7 | 2 | 137 (133, 141) | 8 | 2 | 136 (133, 138) | 35 | 8 | 134 (131, 136) | 14 | 20 | 134 (132, 138) | 8 | 11 |
| Potassium (mmol/L) | 4 (4, 4) | 7 | 2 | 4 (4, 4) | 8 | 2 | 4 (4, 4) | 35 | 8 | 4 (4, 4) | 14 | 20 | 4 (4, 4) | 8 | 11 |
| Urea (mmol/L) | 8 (6, 11) | 7 | 2 | 10 (7, 15) | 8 | 2 | 5 (4, 8) | 35 | 8 | 6 (4, 9) | 14 | 20 | 8 (6, 13) | 8 | 11 |
| Creatinine (umol/L) | 86 (63, 119) | 7 | 2 | 103 (74, 156) | 8 | 2 | 74 (59, 92) | 35 | 8 | 85 (65, 116) | 14 | 20 | 89 (66, 140) | 8 | 11 |
| Oxygen pulse oximetry saturations (%) | 96 (94, 97) | 7 | 2 | 96 (94, 97) | [b] | [b] | 96 (95, 98) | 41 | 9 | 95 (93, 97) | [b] | [b] | 95 (92, 97) | [b] | [b] |
| Fraction Inspired Oxygen (%) | 24 (21, 28) | 77 | 17 | 28 (21, 36) | 42 | 12 | 24 (21, 28) | 143 | 32 | 36 (28, 60) | 13 | 19 | 28 (21, 95) | 12 | 16 |
| Respiratory Rate (breaths per minute) | 19 (18, 20) | 7 | 2 | 20 (18, 24) | [b] | [b] | 19 (18, 20) | 40 | 9 | 22 (20, 28) | [b] | [b] | 21 (18, 26) | [b] | [b] |
| Heart rate (beats per minute) | 84 (73, 95) | 7 | 2 | 88 (76, 101) | [b] | [b] | 88 (78, 101) | 41 | 9 | 96 (83, 105) | [b] | [b] | 88 (76, 102) | [b] | [b] |
| Systolic Blood Pressure (mmHg) | 131 (114, 149) | 7 | 2 | 129 (113, 146) | [b] | [b] | 126 (116, 142) | 44 | 10 | 131 (119, 142) | [b] | [b] | 131 (118, 141) | [b] | [b] |
| Diastolic Blood Pressure (mmHg) | 69 (61, 79) | 7 | 2 | 68 (60, 79) | [b] | [b] | 72 (65, 80) | 44 | 10 | 74 (64, 82) | [b] | [b] | 70 (62, 79) | [b] | [b] |
| Temperature ($^{o}$C) | 36 (36, 37) | 7 | 2 | 37 (36, 37) | [b] | [b] | 37 (36, 38) | 41 | 9 | 37 (37, 38) | [b] | [b] | 37 (62, 79) | [b] | [b] |

ORIGINAL UNEDITED MANUSCRIPT

Abbreviations: Severe Acute Respiratory Syndrome CoronaVirus 2 (SARS-CoV2), Number (No.), Intensive Care Unit (ICU), Interquartile Range (IQR)

[a] Values are expressed as median (interquartile range)

[b] Cells with <5 cases were omitted

ORIGINAL UNEDITED MANUSCRIPT

Table 3. Risk prediction models for next day escalation or death amongst patients eligible for escalation ICU, and for next day mortality amongst patients not eligible for escalation to ICU, between 21 February 2020 until 30 June 2020 (derivation cohort)

| Predictor covariates | Patients not for escalation: Next day death [a] (Total observed patient days = 9,338 days) | | | | Patients for escalation: Next day ICU admission or death [b] (Total observed patient days = 3,275 days) | | | |
|---|---|---|---|---|---|---|---|---|
| | Patients with complete data only | | All patients by imputing missing values | | Patients with complete data only | | All patients by imputing missing values | |
| | Hazard ratios | 95% Confidence intervals | Hazard ratios | 95% Confidence intervals | Hazard ratios | 95% Confidence intervals | Hazard ratios | 95% Confidence intervals |
| Lagged change in daily mean Haemoglobin (g/L) | | | | | 0.99 | 0.98, 0.99 | 0.99 | 0.98,1.00 |
| Log(mean daily Neutrophils) (log $10^9$/L) | 1.58 | 1.24, 2.03 | 1.60 | 1.24, 2.05 | 2.39 | 1.54, 3.71 | 2.11 | 1.35,3.29 |
| log(daily mean Lymphocyte count) (log $10^9$/L) | 0.76 | 0.63, 0.91 | 0.75 | 0.62, 0.91 | 0.57 | 0.39, 0.82 | 0.59 | 0.38,0.91 |
| log(Platelet count) (log $10^9$/L) | 0.69 | 0.54, 0.88 | 0.72 | 0.56, 0.92 | 0.53 | 0.32, 0.89 | 0.49 | 0.28,0.86 |
| Daily mean Sodium (mmol/L) | 1.03 | 1.02, 1.05 | 1.03 | 1.02, 1.05 | | | | |
| Daily mean Potassium (mmol/L) | | | | | 2.51 | 1.61, 3.93 | 1.97 | 1.22,3.18 |
| log(daily mean Urea) (log mmol/L) | 1.56 | 1.24, 1.96 | 1.54 | 1.23, 1.94 | 0.61 | 0.40, 0.93 | 0.56 | 0.34,0.93 |
| Highest daily $FiO_2$ (%) | 1.03 | 1.02, 1.03 | 1.03 | 1.02, 1.03 | 1.04 | 1.03, 1.05 | 1.04 | 1.03,1.05 |
| Daily lowest oxygen saturation (%) | 0.98 | 0.97, 0.99 | 0.98 | 0.97, 0.99 | | | | |
| Highest daily Temperature ($^o$C) | 0.71 | 0.60, 0.85 | 0.71 | 0.60, 0.85 | 1.26 | 1.00, 1.60 | 1.45 | 1.13,1.86 |
| Highest respiratory rate (breaths per minute) | 1.05 | 1.04, 1.06 | 1.05 | 1.04, 1.06 | 1.05 | 1.03, 1.08 | 1.06 | 1.03,1.09 |
| Highest daily heart rate (beats per minute) | 1.02 | 1.01, 1.03 | 1.01 | 1.00, 1.01 | | | | |
| Within day change in heart rate (beats per minute) | 1.02 | 1.01, 1.02 | 1.02 | 1.01, 1.02 | | | | |

ORIGINAL UNEDITED MANUSCRIPT

| | | | | | | | | |
|---|---|---|---|---|---|---|---|---|
| Lagged change in highest daily heart rate (beats per minute) | | | | | 1.02 | 1.02, 1.03 | 1.02 | 1.01,1.03 |
| Age on admission (years) | 1.01 | 0.99, 1.02 | 1.01 | 1.00, 1.02 | 1.11 | 1.03, 1.19 | 1.09 | 0.99,1.19 |
| $Age^2$ on admission ($years^2$) | | | | | 1.00 | 1.00, 1.00 | 1.00 | 1.00,1.00 |

Abbreviations: Intensive Care Unit (ICU)

[a] Patients not for escalation model: log likelihood -1,190, likelihood ratio test 511 (df = 12)

[b] Patients for escalation model: log likelihood -385, likelihood ratio test 287 (df = 12)

ORIGINAL UNEDITED MANUSCRIPT

**Figure Legends**

**Figure 1.** Stacked bar chart of magnitude of daily calculated linear predictor (derived in this study) overlaid with a line plot of the number of patients who were escalated to ICU or died the next day from those who were eligible for escalation (calculated using leave one out cross validation) in: First wave in which score was derived, Nottingham, UK, 21 February 2020 until 30 June 2020

**Figure 2.** Stacked bar chart of magnitude of daily calculated linear predictor (derived in this study) overlaid with a line plot of the number of patients who died each day from those who were ineligible for escalation to ICU (calculated using leave one out cross validation) in: First wave in which score was derived, Nottingham, UK, 21 February 2020 until 30 June 2020

**Figure 3.** Stacked bar chart of magnitude of daily calculated linear predictor (derived in this study) overlaid with a line plot of the number of patients who were escalated to ICU or died the next day from those who were eligible for escalation (calculated using leave one out cross validation) in: Second wave in which score was validated, Nottingham, UK, 1 July 2020 until 31 December 2020

**Figure 4.** Stacked bar chart of magnitude of daily calculated linear predictor (derived in this study) overlaid with a line plot of the number of patients who died each day from those who were ineligible for escalation to ICU (calculated using leave one out cross validation) in: Second wave in which score was validated, Nottingham, UK, 1 July 2020 until 31 December 2020

ORIGINAL UNEDITED MANUSCRIPT

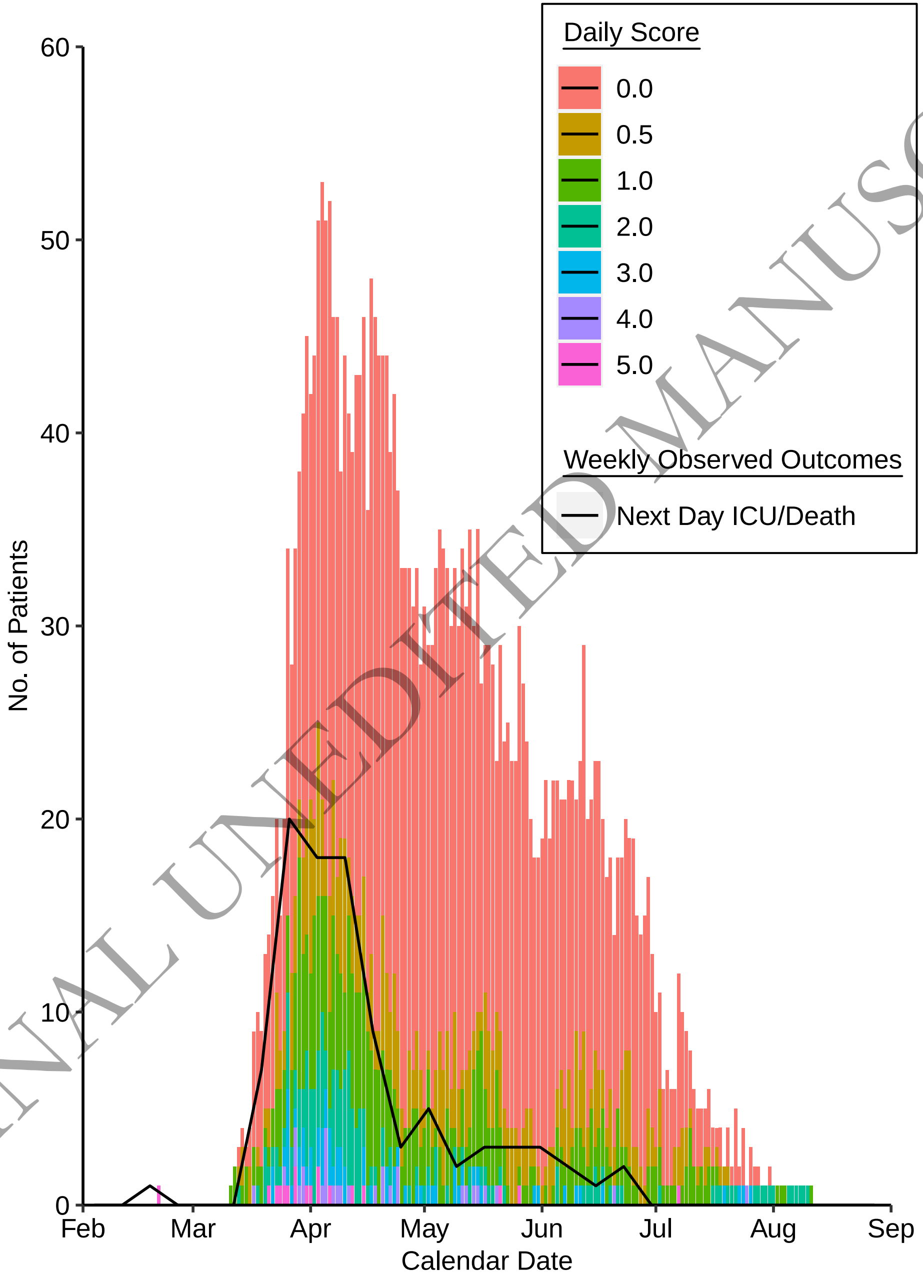

ORIGINAL UNEDITED MANUSCRIPT

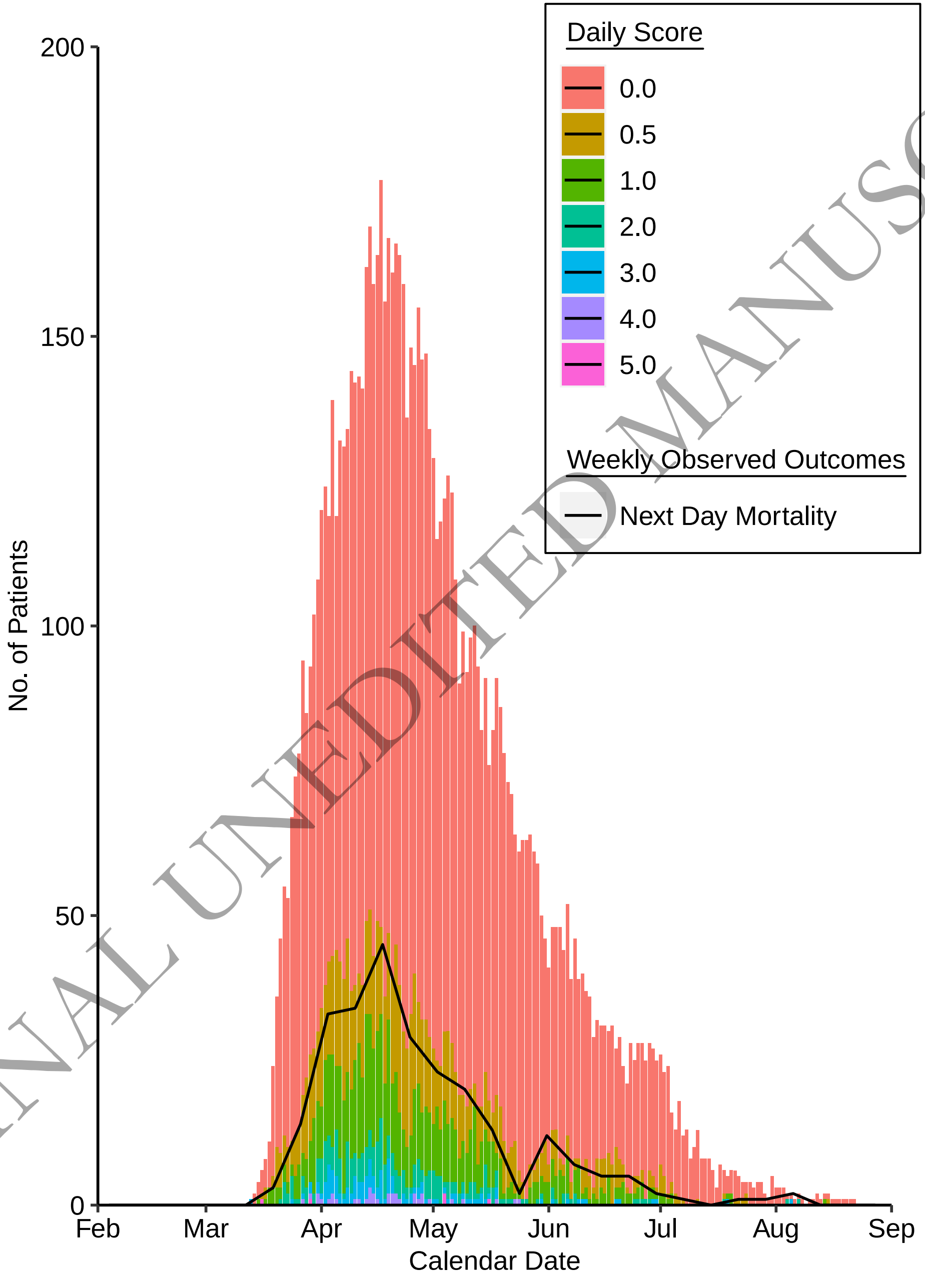

ORIGINAL UNEDITED MANUSCRIPT

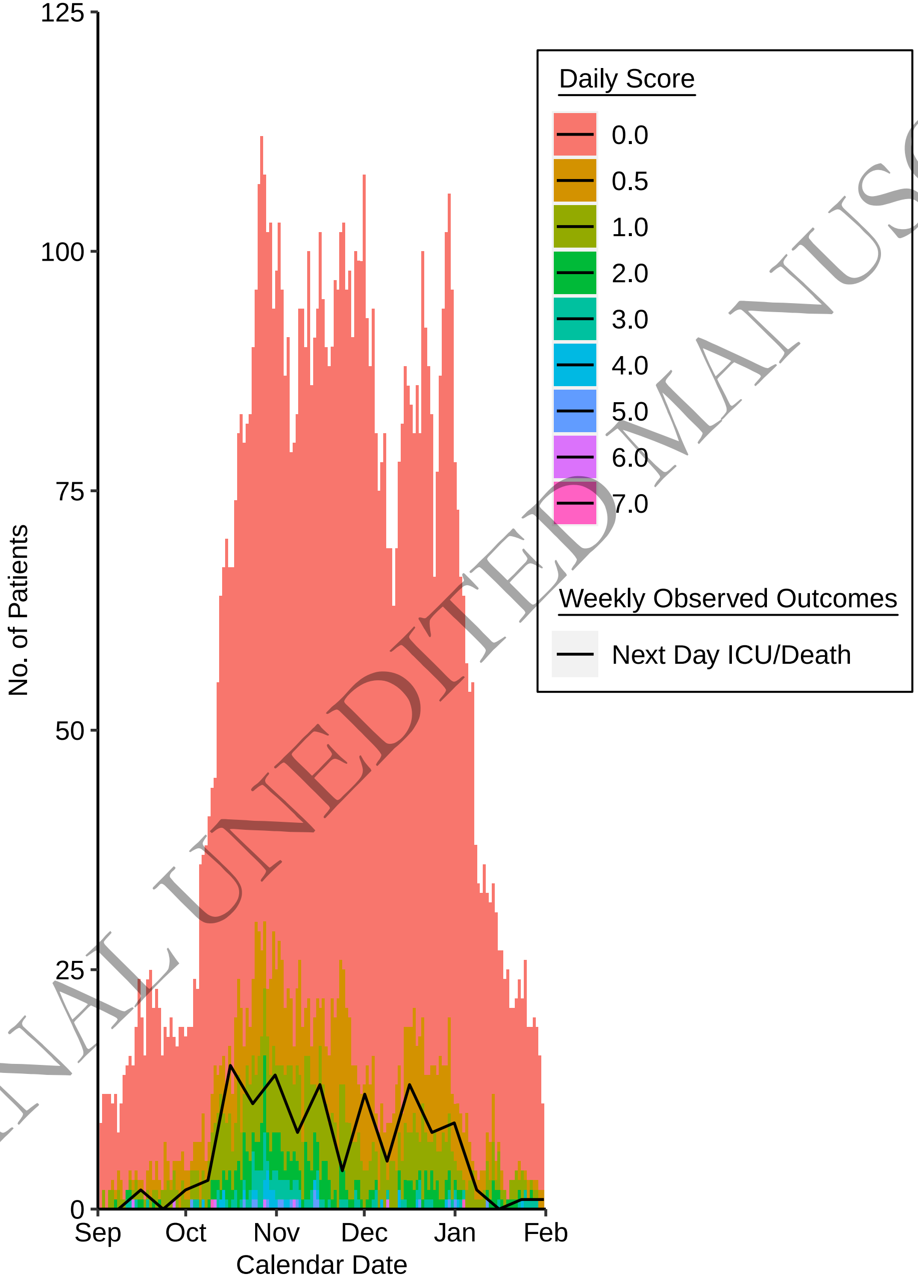

ORIGINAL UNEDITED MANUSCRIPT

INAL UNEDITED MANUSC

## Supplementary Material

Web_Material_kwac126

