# Predicting Need for Escalation of Care or Death From Repeated Daily Clinical Observations and Laboratory Results in Patients With Severe Acute Respiratory Syndrome Coronavirus 2

**DOI:** 10.1093/aje/kwac126

**Published:** 2022-07-22

**Authors:** Colin J Crooks, Joe West, Andrew Fogarty, Joanne R Morling, Matthew J Grainge, Sherif Gonem, Mark Simmonds, Andrea Race, Irene Juurlink, Steve Briggs, Simon Cruickshank, Susan Hammond-Pears, Timothy R Card

**Keywords:** coronavirus disease 2019, COVID-19, critical care, mortality, SARS-CoV-2, severe acute respiratory syndrome coronavirus 2, survival analysis

## Abstract

We compared the performance of prognostic tools for severe acute respiratory syndrome coronavirus 2 (SARS-CoV-2) using parameters fitted either at the time of hospital admission or across all time points of an admission. This cohort study used clinical data to model the dynamic change in prognosis of SARS-CoV-2 at a single hospital center in the United Kingdom, including all patients admitted from February 1, 2020, to December 31, 2020, and then followed up for 60 days for intensive care unit (ICU) admission, death, or discharge from the hospital. We incorporated clinical observations and blood tests into 2 time-varying Cox proportional hazards models predicting daily 24- to 48-hour risk of admission to the ICU for those eligible for escalation of care or death for those ineligible for escalation. In developing the model, 491 patients were eligible for ICU escalation and 769 were ineligible for escalation. Our model had good discrimination of daily risk of ICU admission in the validation cohort (*n* = 1,141; *C* statistic: *C* = 0.91, 95% confidence interval: 0.89, 0.94) and our score performed better than other scores (National Early Warning Score 2, International Severe Acute Respiratory and Emerging Infection Comprehensive Clinical Characterisation Collaboration score) calculated using only parameters measured on admission, but it overestimated the risk of escalation (calibration slope = 0.7). A bespoke daily SARS-CoV-2 escalation risk prediction score can predict the need for clinical escalation better than a generic early warning score or a single estimation of risk calculated at admission.

## Abbreviations

CI: confidence interval
ICU: intensive care unit
IQR: interquartile range
ISARIC4C: International Severe Acute Respiratory and Emerging Infection Comprehensive Clinical Characterisation Collaboration
NEWS2: National Early Warning Score 2
NHS: National Health Service
NUH: Nottingham University Hospitals
PCR: polymerase chain reaction
SARS-CoV-2: severe acute respiratory syndrome coronavirus 2

The severe acute respiratory syndrome coronavirus 2 (SARS-CoV-2) pandemic has brought some health systems to a state of near collapse (1) and has increased the risk of death from other diseases due to the diversion of resources (2, 3).

During the first wave of the pandemic, in 2020, many prognostic scores (4–6) like the International Severe Acute Respiratory and Emerging Infection Comprehensive Clinical Characterisation Collaboration (ISARIC4C) mortality score (5, 7) were created bespoke for SARS-CoV-2 but were based on information from a single time point (hospital admission). However, clinicians make clinical decisions regarding escalation of care throughout the course of disease. Other scores that were used (such as National Early Warning Score 2 (NEWS2)) aimed at more dynamic use throughout the disease course but were not disease-specific (8). A number of these scores performed reasonably, with an area under the receiver operating characteristic curve of 0.77 (95% confidence interval (CI): 0.76, 0.77) for a validation cohort study on ISARIC4C mortality (5) and 0.77 (95% CI: 0.76, 0.78) for a validation cohort study on ISARIC4C deterioration (7), and for NEWS2, the area under the receiver operating characteristic curve varied from 0.623 to 0.815 between hospitals (9).

A score that was both dynamic (i.e., calculated daily using all available clinical measurements) and optimized for SARS-CoV-2 might perform better than the alternatives and might be of greater value to both clinicians and hospital managers. We therefore aimed to derive and validate a disease severity score based on daily clinical observations and blood measurements which would predict next-day intensive care unit (ICU) admission or mortality for those eligible for escalation of care and next-day mortality for those ineligible for escalation. We also planned to compare the performance characteristics of this new score with that of the NEWS2 and ISARIC4C scores.

## METHODS

We carried out and reported this study in accordance with the Transparent Reporting of a Multivariable Prediction Model for Individual Prognosis or Diagnosis (TRIPOD) guidelines (10).

### Study design, setting, and populations

This retrospective, observational cohort study was conducted at Nottingham University Hospitals (NUH) National Health Service (NHS) Trust, a dual-hospital teaching trust in Nottingham, United Kingdom. All admitted patients were identified with a confirmed SARS-CoV-2 diagnosis via either 1) a positive result on polymerase chain reaction (PCR) testing of a nasopharyngeal sample or 2) a recorded clinical diagnosis based on typical radiological features of SARS-CoV-2. We included patients who were diagnosed from February 21, 2020 (the date of disease onset of the first known case at NUH) to June 30, 2020, inclusive, for the derivation cohort and from July 1, 2020, to December 31, 2020, for the validation cohort. All follow-up continued until the earliest date of either discharge from the hospital or the day prior to ICU admission or death, up to February 28, 2021. We split this cohort in order to derive 2 separate models based on the attending physician’s decision as to whether patients were eligible or ineligible for escalation to ICU admission. This decision was made as part of a patient’s routine clinical care based on their frailty and comorbidity. All demographic information and data on comorbid conditions, ceiling-of-care decisions (i.e., the maximum level of critical care support judged appropriate), laboratory tests, and clinical observations were extracted for the identified hospital admissions. Patients entered the derivation cohort for the prediction models from the earliest time at which both clinical observations and blood tests (complete blood count, blood urea nitrogen, and electrolytes) were available after SARS-CoV-2 diagnosis.

### Statistical analysis

#### Outcome.

We defined 2 cohorts. First, for those patients eligible for escalation to respiratory support in the ICU, we defined a combined outcome of either first ICU admission or death within 60 days of the first date of SARS-CoV-2 diagnosis. For those patients who were ineligible for escalation to respiratory support in the ICU, death alone was defined as the outcome.

#### Baseline covariates.

Age was categorized as a linear variable, as a quadratic transformation, and in 20-year categories (20–39, 40–59, 60–79, or ≥80 years), and likelihood ratio tests were used to select the best fit. The presence of comorbidity was categorized by the recording of any comorbidity on the Charlson comorbidity index (11).

#### Time-varying covariates.

We aimed to investigate whether time-varying measures allowed the model to better capture the dynamic changes of risk in comparison with admission-only scores, like ISARIC4C, or scores based on a snapshot of point estimates, like NEWS2. Therefore, daily summary measures of blood tests and observations were derived as follows: 1) the daily mean value of each blood test and the worst daily value for clinical observations, to capture the current magnitude of each measure; 2) the daily change (difference between the first and last measurements within a day), to capture the short-term within-day trend of each measure; and 3) the lagged change in the mean or worst value from the previous day, to capture the longer-term between-day trend. Last observed measurements were carried forward for calculation of the daily summary measures, and patient days prior to measurements’ being available were excluded. These lagged daily summary measures were then used to predict outcomes on the following day. On the first day of admission, when lagged measures were not calculable, the daily measures from that same day (the day of admission) were used.

To assess the effect of excluding patient days prior to measurements’ being available, we performed a sensitivity analysis imputing missing data on the day of admission to derive 30 imputation data sets using multilevel multiple imputations by chained equations with the R package “mice” (12).

#### Model selection and assumptions.

Covariates for a time-varying Cox proportional hazards model were selected using both forward and backward steps with Akaike’s information criterion as a measure of goodness of fit (using the R packages “survival” (13) and “MASS” (14)) and bootstrapping of the process 100 times to assess optimism and the consistency with which parameters were selected. We assessed the proportional hazards assumption by visually examining the Schoenfeld residuals and testing the covariate for a fitted slope versus time. For the missing-data sensitivity analysis, the models were refitted to the imputed data sets, and the results were pooled using Rubin’s rules.

#### Sample size.

Prior to the study, the outcome prevalence was anticipated to be 0.12–0.27, and a lower bound for the new model’s acceptable *R*^2^ value was anticipated to be 0.15. This estimated a sample size of approximately 500 patients using 10 candidate predictors, as shown in Web Table 1 (available at https://doi.org/10.1093/aje/kwac126).

#### Internal validation and comparison with NEWS2 and ISARIC4C.

The performance of the model was tested in the development of the score using the *C* statistic fitted with leave-1-out cross-validation in the derivation cohort across different time points. This was performed by sequentially excluding each patient in turn with all their observations. We then validated the calibration and performance of the score in the second-wave validation cohort using both the *C* statistic and the integrated Brier score (an averaged measure, between 0 and 1, of the difference between observed and predicted survival, adjusted for censoring and time-varying covariates (15)). Finally, we compared our score with the performance of NEWS2 and ISARIC4C, implemented using the published methods. A further sensitivity analysis was undertaken validating the use of only those patients with confirmatory PCR tests for SARS-CoV-2.

All analyses were performed using the R programming language, version 4.0.3 (R Foundation for Statistical Computing, Vienna, Austria). Approval for this work was granted by an NUH Clinical Effectiveness Team audit, the NUH Caldicott Guardian (Data Protection Impact Assessment), and the NHS Health Research Authority (research study ethics approval) Integrated Research Application System.

## RESULTS

Combined (derivation and validation cohorts) demographic characteristics, baseline characteristics, and mortality outcomes are shown in Table 1. Overall, 3,898 patients were admitted to one of the 2 NUH hospitals, and the key differences apparent between the first- and second-wave cohorts were that in the second wave of the pandemic, the median age was slightly lower (76 years in the first wave vs. 72 years in the second wave) and 30-day mortality was substantially lower (25% in the first wave vs. 20% in the second wave).

**Table 1:** Sociodemographic and Other Characteristics (Upon Hospital Admission) of a Derivation Cohort With the Earliest Date of Confirmed SARS-CoV-2 Diagnosis Between February 21, 2020, and June 30, 2020, and a Second-Wave Validation Cohort With the Earliest Date of Confirmed SARS-CoV-2 Diagnosis Before December 31, 2020 (Followed Up Until January 31, 2021), Nottingham, United Kingdom

**Cohort Characteristic**	**Derivation Cohort** **(Admission Before June 30, 2020;** ***n* = 1,443)**			**Validation Cohort** **(Admission After July 1, 2020;** ***n* = 2,455)**		
	**No.**	**%**	**Median (IQR)**	**No.**	**%**	**Median (IQR)**
Age, years			76 (61–85)			72 (54–83)
Male sex	751	52		1,255	51	
Ethnic group						
Other or not stated	255	18		491	20	
Black/mixed	55	4		77	3	
Indian/Pakistani	56	4		144	6	
White	1,077	75		1,743	71	
30-day mortality	365	25		498	20	
Died outside of hospital	41	3		42	2	
30-day ICU admission	151	10		258	11	
Length of stay, days			8 (3–16)			9 (3–20)
Eligible for ICU escalation/CPR	620	43		1,422	58	
NEWS2 score			3 (2–5)			3 (1–4)
ISARIC4C score			10 (7–12)			9 (5–11)
Body mass index^a^						
<20	259	18		393	16	
>30	382	26		671	27	
Tobacco smoking	160	11		313	13	
Vaping	67	5		147	6	
Hazardous alcohol risk^b^	202	14		358	15	
Charlson comorbidity index score			2 (1–3)			1 (0–3)

Abbreviations: CPR, cardiopulmonary resuscitation; ICU, intensive care unit; IQR, interquartile range; ISARIC4C, International Severe Acute Respiratory and Emerging Infection Comprehensive Clinical Characterisation Collaboration; NEWS2, National Early Warning Score 2; SARS-CoV-2, severe acute respiratory syndrome coronavirus 2.

^a^ Weight (kg)/height (m)^2^.

^b^ Patients identified during routine nurse screening assessment at admission as drinking above government-recommended levels of 14 units per week (32).

### First-wave (derivation) cohort

From February 21, 2020, to June 30, 2020, a total of 1,443 patients were admitted to a NUH hospital with clinically confirmed SARS-CoV-2. The daily status of these patients is shown in Web Figure 1 by day of disease course (measured from the day on which SARS-CoV-2 was first recorded).

Of those patients in the derivation cohort, 1,040 (72%) had a confirmatory PCR test, with the remainder having a clinical diagnosis made from typical radiological features (Web Table 2). A total of 491 patients were eligible for escalation of respiratory support with both blood tests and observations recorded during their admission in the time before any escalation to the ICU or death (Web Figure 2A). Ninety of these patients were escalated to ICU care or died while an inpatient within 60 days of the first diagnosis date for derivation of the “eligible for escalation to ICU” model. For derivation of the “ineligible for escalation to ICU” model, 769 patients had observations and blood tests available after the earliest SARS-CoV-2 diagnosis date (Web Figure 2A).

### Second-wave (validation) cohort

From July 1, 2020, to December 31, 2020, a total of 2,455 patients were admitted with a clinical SARS-CoV-2 diagnosis (2,048 with positive PCR tests). Of these patients, 1,356 were eligible for escalation to ICU care and 1,032 were ineligible for escalation (Web Figure 2B).

### Patients eligible for escalation in the first wave: predicting daily risk of next-day ICU admission or death

Table 2 shows initial measurements and missing data for patients at the earliest time point after diagnosis of SARS-CoV-2 when both blood and clinical observations were available. Web Figure 3 shows how selected observations and blood results then varied during the admission stratified by patients’ final outcomes.

**Table 2:** Initial Blood Test Results and Observational Data on 1,391 Patients With Confirmed SARS-CoV-2 and Inpatient Time Observed Prior to Any Escalation to ICU Care, According to Eligibility for Escalation to ICU Care and Worst Outcome Within 60 Days of Diagnosis (Derivation Cohort), Nottingham, United Kingdom, February 21, 2020–June 30, 2020^a^

	**Worst Outcome Within 60 Days of SARS-CoV-2 Diagnosis**														
	**Not Eligible for Escalation to ICU Care**						**Eligible for Escalation to ICU Care**								
	**Survived (*n* = 461)**			**Died (*n* = 345)**			**Not Admitted to ICU (*n* = 440)**			**Admitted to ICU (*n* = 70)**			**Died (*n* = 75)**		
**Blood Test or Clinical Observation**		**Not Measured**			**Not Measured**			**Not Measured**			**Not Measured**			**Not Measured**	
	**Median(IQR)**	**No.**	**%**	**Median(IQR)**	**No.**	**%**	**Median(IQR)**	**No.**	**%**	**Median(IQR)**	**No.**	**%**	**Median(IQR)**	**No.**	**%**
Hemoglobin concentration, g/L	120 (106–133)	7	2	117 (102–136)	10	3	128 (114–143)	34	8	133 (114–145)	13	19	120 (104–136)	8	11
Platelet count, 10^9^ cells/L	236 (177–295)	7	2	214 (162–295)	10	3	233 (176–311)	34	8	227 (182–333)	16	23	226 (158–327)	9	12
Neutrophil count, 10^9^ cells/L	6.1 (4.0–9.1)	7	2	6.7 (4.7–9.9)	10	3	5.1 (3.4–7.8)	34	8	6.4 (4.6–9.1)	13	19	6.8 (4.5–10.9)	8	11
Lymphocyte count, 10^9^ cells/L	0.9 (0.6–1.4)	7	2	0.8 (0.5–1.1)	10	3	1.1 (0.8–1.6)	34	8	0.9 (0.7–1.1)	16	23	0.9 (0.6–1.3)	9	12
Sodium concentration, mmol/L	136 (133–139)	7	2	137 (133–141)	8	2	136 (133–138)	35	8	134 (131–136)	14	20	134 (132–138)	8	11
Potassium concentration, mmol/L	4 (4–4)	7	2	4 (4–4)	8	2	4 (4–4)	35	8	4 (4–4)	14	20	4 (4–4)	8	11
BUN concentration, mmol/L	8 (6–11)	7	2	10 (7–15)	8	2	5 (4–8)	35	8	6 (4–9)	14	20	8 (6–13)	8	11
Creatinine concentration, μmol/L	86 (63–119)	7	2	103 (74–156)	8	2	74 (59–92)	35	8	85 (65–116)	14	20	89 (66–140)	8	11
Oxygen pulse oximetry saturation, %	96 (94–97)	7	2	96 (94–97)	—^b^	—	96 (95–98)	41	9	95 (93–97)	—	—	95 (92–97)	—	—
Fraction of inspired oxygen, %	24 (21–28)	77	17	28 (21–36)	42	12	24 (21–28)	143	32	36 (28–60)	13	19	28 (21–95)	12	16
Respiration rate, breaths/minute	19 (18–20)	7	2	20 (18–24)	—	—	19 (18–20)	40	9	22 (20–28)	—	—	21 (18–26)	—	—
Heart rate, beats/minute	84 (73–95)	7	2	88 (76–101)	—	—	88 (78–101)	41	9	96 (83–105)	—	—	88 (76–102)	—	—
Systolic blood pressure, mm Hg	131 (114–149)	7	2	129 (113–146)	—	—	126 (116–142)	44	10	131 (119–142)	—	—	131 (118–141)	—	—
Diastolic blood pressure, mm Hg	69 (61–79)	7	2	68 (60–79)	—	—	72 (65–80)	44	10	74 (64–82)	—	—	70 (62–79)	—	—
Temperature, °C	36 (36–37)	7	2	37 (36–37)	—	—	37 (36–38)	41	9	37 (37–38)	—	—	37 (62–79)	—	—

Abbreviations: BUN, blood urea nitrogen; ICU, intensive care unit; IQR, interquartile range; SARS-CoV-2, severe acute respiratory syndrome coronavirus 2.

^a^ Blood tests and observations conducted after ICU admission were excluded. The IQR for the time from first confirmed SARS-CoV-2 diagnosis to the time at which clinical observations, complete blood counts, and electrolyte blood tests were all available was ≤25 hours.

^b^ Cells with fewer than 5 cases were omitted.

Modeling daily summary measures of complete blood count, blood urea nitrogen, and electrolyte levels and observations as described in the Methods section showed that a model with both quadratic and linear terms for age had a statistically better fit than a categorical model (likelihood ratio test: *P* = 0.03). Blood cell counts were transformed to the log scale due to positive skew.

The final selected model predicting next-day escalation or death (Table 3) had an overall concordance (*C* statistic) of 0.91 (95% CI: 0.87, 0.94). The adjustment for optimism using the bootstrapped uniform shrinkage factor was estimated at *C* = 0.70 (interquartile range (IQR), 0.62–0.82). The sensitivity analysis imputing missing data attenuated but did not substantially alter the covariates (Table 3). The integrated Brier score confirmed a low mean squared error of 0.01. Residual plots testing the proportional hazards assumption are shown in Web Figure 4 and Web Table 3.

**Table 3:** Hazard Ratios (Risk Prediction Models) for Next-Day Escalation to ICU Admission or Death Among SARS-CoV-2 Patients Eligible for Escalation to ICU Care and for Next-Day Mortality Among Patients Not Eligible for Escalation to ICU Care (Derivation Cohort), Nottingham, United Kingdom, February 21, 2020–June 30, 2020

	**Patients Not Eligible for Escalation: Next-Day Death** ^a^ **(Total Observed Patient Days = 9,338)**				**Patients Eligible for Escalation: Next-Day ICU Admission or Death** ^b^ **(Total Observed Patient Days = 3,275)**			
	**Patients With Complete Data Only**		**All Patients (by Imputing Missing Values)**		**Patients With Complete Data Only**		**All Patients (by Imputing Missing Values)**	
**Predictor Covariate**	**HR**	**95% CI**	**HR**	**95% CI**	**HR**	**95% CI**	**HR**	**95% CI**
Lagged change in daily mean hemoglobin concentration, g/L					0.99	0.98, 0.99	0.99	0.98, 1.00
Log(daily mean neutrophil count), log 10^9^ cells/L	1.58	1.24, 2.03	1.60	1.24, 2.05	2.39	1.54, 3.71	2.11	1.35, 3.29
Log(daily mean lymphocyte count), log 10^9^ cells/L	0.76	0.63, 0.91	0.75	0.62, 0.91	0.57	0.39, 0.82	0.59	0.38, 0.91
Log(platelet count), log 10^9^ cells/L	0.69	0.54, 0.88	0.72	0.56, 0.92	0.53	0.32, 0.89	0.49	0.28, 0.86
Daily mean sodium concentration, mmol/L	1.03	1.02, 1.05	1.03	1.02, 1.05				
Daily mean potassium concentration, mmol/L					2.51	1.61, 3.93	1.97	1.22, 3.18
Log(daily mean BUN concentration), log mmol/L	1.56	1.24, 1.96	1.54	1.23, 1.94	0.61	0.40, 0.93	0.56	0.34, 0.93
Highest daily FiO_2_ concentration, %	1.03	1.02, 1.03	1.03	1.02, 1.03	1.04	1.03, 1.05	1.04	1.03, 1.05
Daily lowest oxygen saturation, %	0.98	0.97, 0.99	0.98	0.97, 0.99				
Highest daily temperature, °C	0.71	0.60, 0.85	0.71	0.60, 0.85	1.26	1.00, 1.60	1.45	1.13, 1.86
Highest respiration rate, breaths/minute	1.05	1.04, 1.06	1.05	1.04, 1.06	1.05	1.03, 1.08	1.06	1.03, 1.09
Highest daily heart rate, beats/minute	1.02	1.01, 1.03	1.01	1.00, 1.01				
Within-day change in heart rate, beats/minute	1.02	1.01, 1.02	1.02	1.01, 1.02				
Lagged change in highest daily heart rate, beats/minute					1.02	1.02, 1.03	1.02	1.01, 1.03
Age upon admission, years	1.01	0.99, 1.02	1.01	1.00, 1.02	1.11	1.03, 1.19	1.09	0.99, 1.19
Age^2^ upon admission, years^2^					1.00	1.00, 1.00	1.00	1.00, 1.00

Abbreviations: BUN, blood urea nitrogen; CI, confidence interval; df, degrees of freedom; FiO_2_, fraction of inspired oxygen; HR, hazard ratio; ICU, intensive care unit; SARS-CoV-2, severe acute respiratory syndrome coronavirus 2.

^a^ “Patients not eligible for escalation” model: model log likelihood = −1,190; likelihood ratio test (full model vs. null model) χ^2^ = 511 (12 df), *P* < 0.0001.

^b^ “Patients eligible for escalation” model: model log likelihood = −385; likelihood ratio test (full model vs. null model) χ^2^ = 287 (12 df), *P* < 0.0001.

Concordance did not alter with cross-validation using bootstrapped samples (*C* = 0.90 (IQR, 0.88–0.91)) and remained high across the follow-up time (Web Figure 5). The corresponding discrimination was lower for both the ISARIC4C mortality score (*C* = 0.64, 95% CI: 0.58, 0.69) and the NEWS2 score (*C* = 0.86, 95% CI: 0.82, 0.90). Restricting the population to just those who had a SARS-CoV-2 positive PCR test did not alter the discrimination (*C* = 0.89 (IQR, 0.87–0.91)). The final algorithm is shown in Web Table 4.

### Patients ineligible for escalation in the first wave: predicting next-day mortality

For patients not eligible for escalation to the ICU, a separate model was built predicting only next-day mortality (Table 3). The model’s discrimination in the derivation cohort with leave-1-out cross-validation using bootstrapped samples was 0.86 (IQR, 0.84–0.89), and it remained high throughout follow-up (Web Figure 6). Baseline survival plots are shown in Web Figure 7. The final algorithm is shown in Web Table 3. The integrated Brier score confirmed a low mean squared error of 0.04. Residual plots testing the proportional hazards association are shown in Web Figure 8 and Web Table 5.

### Calibration and comparison with existing scores in the first wave

**Figure 1:**
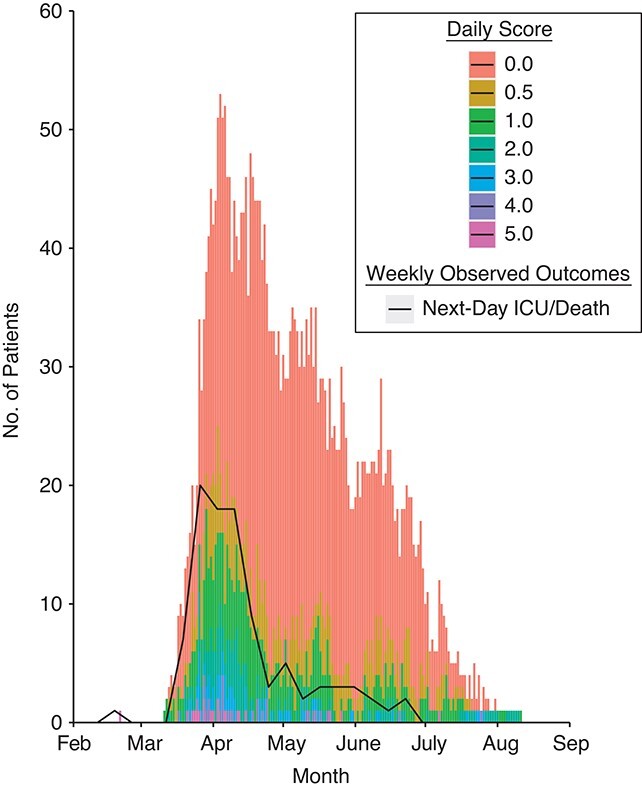
Magnitude of the daily calculated linear predictor score derived in this study (stacked bar chart) overlaid with the number of patients who were escalated to intensive care unit (ICU) admission or died the next day (line plot) among those who were eligible for escalation (calculated using leave-1-out cross-validation) during the first wave of severe acute respiratory syndrome coronavirus 2 infection, in which the score was derived, Nottingham, United Kingdom, February 21, 2020–June 30, 2020.

**Figure 2:**
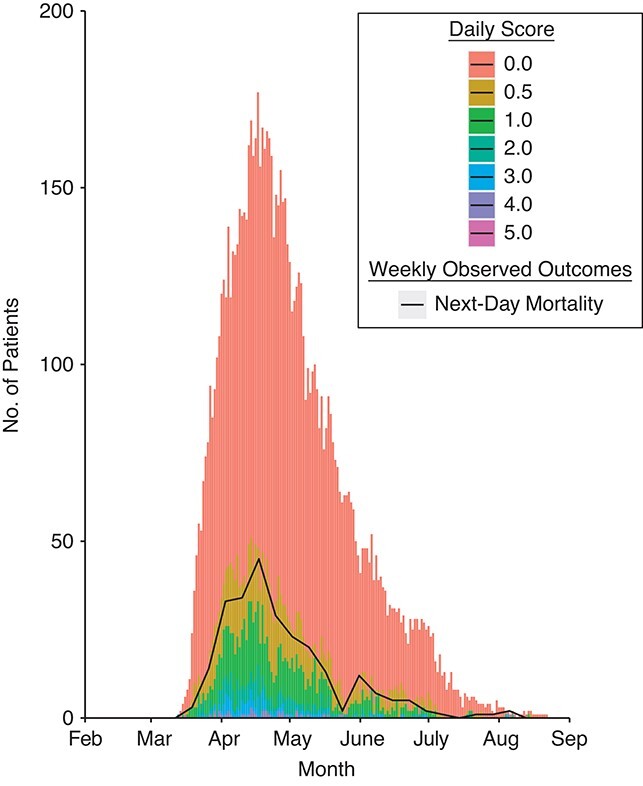
Magnitude of the daily calculated linear predictor score derived in this study (stacked bar chart) overlaid with the number of patients who died each day (line plot) among those who were ineligible for escalation to intensive care unit admission (calculated using leave-1-out cross-validation) during the first wave of severe acute respiratory syndrome coronavirus 2 infection, in which the score was derived, Nottingham, United Kingdom, February 21, 2020–June 30, 2020.

The magnitudes of the 2 derived scores tracked the observed outcomes for inpatients eligible for the ICU (Figure 1) and ineligible for the ICU (Figure 2) in the derivation first-wave cohort.

### Second-wave validation

For patients eligible for escalation to the ICU in the second-wave validation cohort, the discrimination of our score remained high, with a concordance of 0.91 (95% CI: 0.89, 0.94) (Web Figure 9A), and, to a lesser extent, discrimination was also high for the ISARIC4C mortality score (*C* = 0.70, 95% CI: 0.66, 0.75) and NEWS2 (*C* = 0.89, 95% CI: 0.86, 0.92). The integrated Brier score confirmed that the mean squared error remained low at 0.01.

For assessment of calibration, Web Figure 9 shows that the derived score overestimated next-day escalation in the second-wave validation cohort, with a calibration slope of 0.68. In Web Table 6, the negative predictive value remained above 98% for all levels of the derived score, and the positive predictive value was over 40% when the linear predictor was above 4.

For patients ineligible for escalation to ICU care, the model predicting next-day mortality had a discrimination of 0.88 (95% CI: 0.86, 0.89) and a calibration slope of 0.69 (Web Figure 9B). In comparison, the discrimination of the daily NEWS2 score for next-day mortality was 0.81 (95% CI: 0.78, 0.83), and that of the ISARIC4C score was 0.79 (95% CI: 0.76, 0.81). In Web Table 6, the negative predictive value remained above 97% for all levels of the derived score, and the positive predictive value was 60% when the linear predictor was above 4. The integrated Brier score showed that the mean squared error was higher at 0.05.

**Figure 3:**
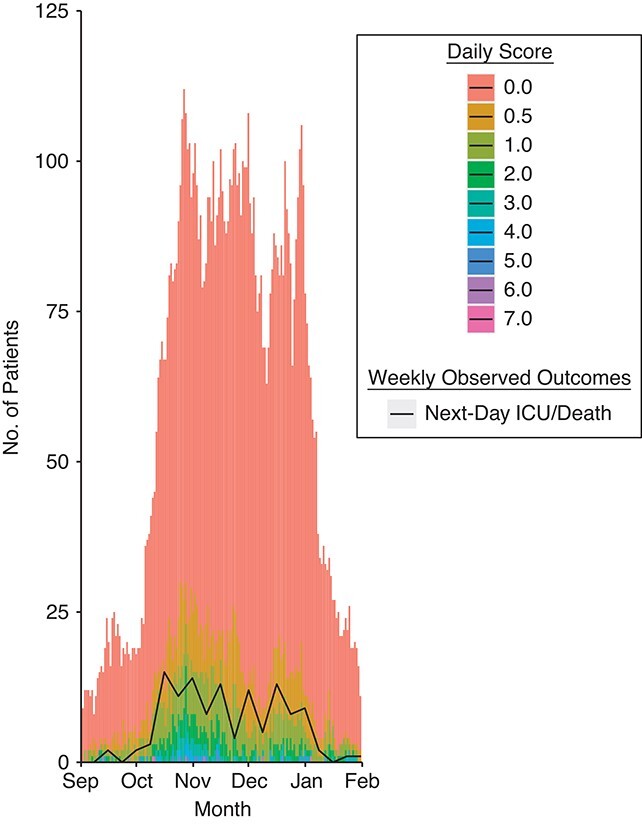
Magnitude of the daily calculated linear predictor score derived in this study (stacked bar chart) overlaid with the number of patients who were escalated to intensive care unit (ICU) admission or died the next day (line plot) among those who were eligible for escalation to intensive care unit (ICU) admission during the second wave of severe acute respiratory syndrome coronavirus 2 infection, in which the score was validated, Nottingham, United Kingdom, July 1, 2020–December 31, 2020.

**Figure 4:**
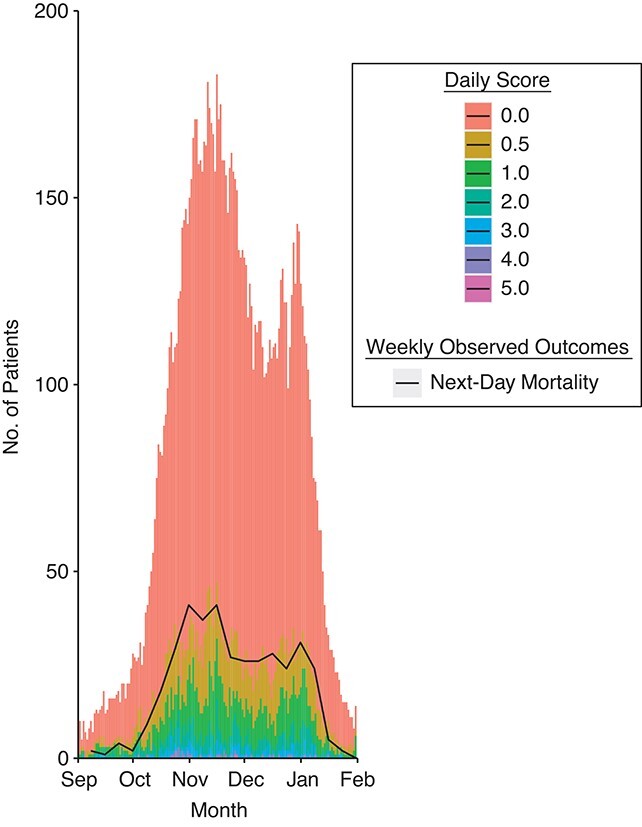
Magnitude of the daily calculated linear predictor score derived in this study (stacked bar chart) overlaid with the number of patients who died each day (line plot) among those who were ineligible for escalation to intensive care unit admission during the second wave of severe acute respiratory syndrome coronavirus 2 infection, in which the score was validated, Nottingham, United Kingdom, July 1, 2020–December 31, 2020.

The magnitude of the 2 derived scores tracked the observed outcomes for inpatients eligible (Figure 3) and ineligible (Figure 4) for the ICU in the second-wave validation cohort, but at lower thresholds than the derivation cohort, reflecting the change in calibration.

## DISCUSSION

### Main findings

This study incorporated daily clinical and laboratory measurements with baseline characteristics to predict the daily dynamic risk of next-day escalation of care or mortality in patients with SARS-CoV-2 with better precision throughout the hospital stay than using the same parameters from a model derived only from measurements taken at admission. The validation showed excellent discrimination and accuracy (as measured by the integrated Brier score), but it overpredicted death and escalation at the thresholds taken from the derivation cohort. This is likely to reflect the change in demographic characteristics and clinical practice between the first and second UK waves of the SARS-CoV-2 pandemic, given changes in escalation practice (16, 17) and the introduction of the use of steroids (18, 19). Our results suggest that using a dynamic score derived from daily blood and clinical measurements could provide better prediction of the need for escalation of care in SARS-CoV-2 than scores derived from similar parameters measured at a single time point (i.e., on admission).

### Strengths and weaknesses

Our study included all patients who were admitted to one of 2 large teaching hospitals in the Midlands region of the United Kingdom serving a population that covers metropolitan, suburban, and rural areas throughout an 8-month period of 2020. The richness and uniformity of our data was a strength of a single-center study, but it was gained at the cost of limiting our analyses to 1 organization and therefore the decisions of 1 cohort of clinicians. This leads to questions regarding generalizability which can only be answered by external validation. However, the diverse population of Nottingham as a representative cross-section of the UK population and the standardization of care across the NHS suggest that our findings will be replicable.

Through our use of electronic patient record systems, we had access to comprehensive data on sociodemographic, clinical, and laboratory variables, including all measurements recorded electronically through the patient’s admission. We also had available complete follow-up data for escalation of care, death (including out-of-hospital death), and discharge from the hospital for 60 days after admission, and (importantly) there was therefore little bias due to missing outcomes, loss to follow-up, or other common biases seen with observational cohort studies.

The missing exposure data that were observed in the cohorts reflects clinical decision-making—for example, patients who were frail and so received compassionate care without the imposition of blood tests and observations, patients who were too well to be kept in the hospital for blood tests and observations, and patients whose treatment was escalated upon admission and so did not have measurements available for the pre-event observation time. Therefore, the missing data were not missing at random, and this is demonstrated by the attenuation of some of the associations in the multiple-imputation sensitivity analysis. For our implementation locally, we used only the model derived from patients with clinical observations and blood tests available in the pre-escalation period, since this had the most clinical relevance for patients being actively managed with clinical equipoise in their care.

Following the development and validation of our score in 2020, there were many developments in the management of SARS-CoV-2, including new treatments (for which we do not have electronic recording), vaccinations (which began after follow-up of our study validation cohort ended in 2021), and new SARS-CoV-2 variants (which did not reach significant levels in the United Kingdom during the 2020 study period). Trigger thresholds for severe disease from our score should therefore be monitored and updated locally, depending on the patient population and setting, as shown in our calibration results. However, throughout 2021, our score, as presented in this paper, has continued to correctly discriminate between patients who have more severe disease and those who have less severe disease. Ongoing auditing of the score’s implementation within our hospital trust for the first 10 months of 2021 (to allow complete 60-day follow-up) showed that discrimination remained high (*C* = 0.92, as measured by the *C* statistic for next-day ICU admission), as compared with NEWS2 (*C* = 0.86) and ISARIC4C (*C* = 0.67). This demonstrated that clinical markers of severity remained the same for patients who became sick, while changes in vaccination, variants, and treatment might reduce the number of people reaching those markers of severity.

### Interpretation

Our study is best compared with other large population-based studies from single cities or regions around the world that have reported their experience through the SARS-CoV-2 pandemic (20–27) and the relevant UK studies (28–30). The distribution of sociodemographic risk factors and their association with poor prognosis with respect to age and sex is similar to that of those studies. Our risk prediction model is unique in using longitudinal daily clinical and laboratory measures to estimate the need for next-day escalation of care or the risk of death. In that respect, we cannot compare it directly with other published risk models, but in relation to those derived within UK populations it performs better (5, 7, 9, 30), and for the reasons stated above it is at low risk of bias. In particular, compared with the robustly developed ISCARIC4C mortality prediction score (5) and the ISARIC4C deterioration score (7), our model performs better on a daily basis—showing the value of incorporating repeated measurements of clinical observations and blood results into the prediction of prognosis for patients admitted to the hospital with SARS-CoV-2. We currently use these models, integrated into the data warehouse within our hospital, to provide a live dynamic overview of the coronavirus disease 2019 inpatient cohort by current severity (as opposed to admission severity identified by other scores) and to identify locations within the hospital with higher burdens of severe coronavirus disease 2019.

### Conclusions

We have shown that incorporating daily measurements of clinical observations and blood tests improves the accuracy of the prediction of prognosis in secondary-care patients with SARS-CoV-2 in comparison with similar scoring systems that are based on the use of data from only a single point in time.

## Supplementary Material

Web_Material_kwac126
