# Supplementary material for: Predicting Need for Escalation of Care or Death From Repeated Daily Clinical Observations and Laboratory Results in Patients With Severe Acute Respiratory Syndrome Coronavirus 2
Source: Am J Epidemiol. 2022 Jul 22;191(11):1944–53. doi: 10.1093/aje/kwac126 (PMC9384527; doi:10.1093/aje/kwac126)
Supplement: Web_Material_kwac126 [file web_material_kwac126.pdf]

WEB MATERIAL

Predicting Need for Escalation of Care or Death From Repeated Daily Clinical Observations and Laboratory Results in Patients With Severe Acute Respiratory Syndrome Coronavirus 2

Colin J. Crooks, Joe West, Andrew Fogarty, Joanne R. Morling, Matthew J. Grainge, Sherif Gonem, Mark Simmonds, Andrea Race, Irene Juurlink, Steve Briggs, Simon Cruickshank, Susan Hammond-Pears, and Timothy R. Card

Contents:

Web Table 1 2
Web Table 2 3
Web Table 3 4
Web Table 4 5
Web Table 5 6
Web Table 6 7
Web Figure 1 8
Web Figure 2A 9
Web Figure 2B 9
Web Figure 3 10
Web Figure 4 17
Web Figure 5 18
Web Figure 6: 19
Web Figure 7 20
Web Figure 8 21
Web Figure 9 22

Web Table 1
Range of sample size calculations performed

		Calculated using pmsampsize command in Stata (Riley, RD, Snell, KIE, Ensor, J, et al. Minimum sample size for developing a multivariable prediction model: PART II - binary and time-to-event outcomes. *Statistics in Medicine*. 2019; 38: 1276– 1296. https://doi.org/10.1002/sim.7992)		
		Minimum sample size to meet requirements with 10 parameters in model and model r2 = 0.15		
Prevalence	Expected number of events	Global shrinkage (0.9)	Difference between r2 and adjusted r2 <0.05	Precision of average outcome risk +/- 0.05
0.12	66	549	349	163
0.15	83	549	318	198
0.18	99	549	298	227
0.21	116	549	283	255
0.24	132	549	270	281
0.27	149	549	263	303
Prevalence		Global shrinkage (0.89)	Difference between r2 and adjusted r2 <0.05	Precision of average outcome risk +/- 0.05
0.12	60	493	349	163
0.15	74	493	318	198
0.18	89	493	298	227
0.21	104	493	283	255
0.24	119	493	270	281
0.27	134	493	263	303

Web Table 2

Sociodemographic and other characteristics on admission of the cohort who were admitted to hospital with SARS-COV-2 21 February 2020 until 30 June 2020

	Admission: Clinical diagnosis only	Admission: PCR positive
N	403	1040
Age (years) median (IQR)	77 (62, 85)	76 (60, 84)
Male	198 (49%)	553 (53%)
Other or not stated ethnic group	58 (14%)	197 (19%)
Black/Mixed ethnic group	10 (2%)	45 (4%)
Indian/Pakistani ethnic group	9 (2%)	47 (5%)
White ethnic group	326 (81%)	751 (72%)
30 day mortality	83 (21%)	282 (27%)
Died out of hospital	13 (3%)	28 (3%)
30 day ICU admission	21 (5%)	130 (12%)
Length of stay, days (IQR)	8 (4, 13)	8 (3, 18)
For escalation/CPR	153 (38%)	467 (45%)
NEWS2, median (IQR)	3 (2, 5)	3 (2, 5)
ISARIC-4C, median (IQR)	10 (7, 12)	10 (6, 12)
BMI <20, n, %	75 (19%)	184 (18%)
BMI >30, n, %	93 (23%)	289 (28%)
Smoking, n, %	73 (18%)	87 (8%)
Vaping, n, %	27 (7%)	40 (4%)
Alcohol risk, n, %	57 (14%)	145 (14%)
Charlson Index, median (IQR)	2 (1, 4)	2 (0, 3)

Web Table 3

Test for slope of zero for Schoenfeld residuals versus time for next day ICU escalation model

Model of risk of next day escalation to ICU	*Test for slope of zero for Schoenfeld residuals versus time:*
	p value (Chi2)
Lagged change in daily mean Haemoglobin	0.02
log(Neutrophil count)	0.94
log(Lymphocyte count)	0.66
Log(Daily mean platelet count)	0.83
Daily mean Potassium	0.83
Lagged change in daily mean Urea	0.53
FiO2 (%)	0.20
Highest Temperature (^{o}C)	0.0003
Lagged change in daily highest heart rate	0.21
Highest daily respiratory rate	0.22

Web Table 4
Calculation of linear predictors for models used for validation in second wave

Linear predictor for next day escalation/death model for patients eligible for escalation =<br>-0.0125674 * (lag_Haemoglobin - 9.213636) +<br>0.8701899 * (log_Neutrophils - 1.537172) +<br>-0.5621189 * (log_Lymphocytes - 0.083262) +<br>-0.6348783 * (log_PlateletCount - 5.484431) +<br>0.9202828 * (Potassium - 4.132611) +<br>-0.4942963 * (log_Urea - 1.731216) +<br>0.2340765 * (TEMPERATURE - 37.189060) +<br>0.0531105 * (RESP_RATE - 21.033282) +<br>0.0225751 * (lag_HEART_RATE - 6.655470) +<br>0.0398151 * (FiO2 - 28.850724) +<br>0.1032351 * (AGE_AT_ADMISSION - 57.969800) +<br>-0.0007869 * (AGE_AT_ADMISSION^2 - 3824.085054)
Linear predictor for next day death model for patients ineligible for escalation =<br>0.4574248 * (log_Neutrophils - 1.6873937) +<br>-0.2822816 * (log_Lymphocytes - -0.0250936) +<br>-0.3771835 * (log_PlateletCount - 5.4290112) +<br>0.0330612 * (Sodium - 137.3990841) +<br>0.4446858 * (log_Urea - 2.0373875) +<br>-0.3360895 * (TEMPERATURE - 36.99199) +<br>0.0494824 * (RESP_RATE - 20.8943202) +<br>0.0198026 * (HEART_RATE - 90.2390630) +<br>0.0164881 * (delta_HEART_RATE - -0.1344529) +<br>0.0251410 * (FiO2 - 30.3385603) +<br>-0.0215520 * (sO2 - 93.3695048) +<br>0.0094745 * (AGE_AT_ADMISSION - 79.9594609)

Web Table 5

Test for slope of zero for Schoenfeld residuals versus time for next day mortality model

Model of risk of next day mortality	*Test for slope of zero for Schoenfeld residuals versus time:*
	p value (Chi2)
log(Daily mean Neutrophil count)	0.65
log(Daily mean Lymphocyte count)	0.48
log(Daily mean Platelet count)	0.86
Daily mean Sodium	0.25
log(Daily mean Urea)	0.21
Highest FiO2 (%)	0.21
Lowest daily Oxygen saturations (%)	0.07
Daily highest Temperature	0.19
Daily highest respiratory rate	0.12
Daily highest heart rate	0.20
Within day change in heart rate	0.04

Web Table 6
Positive and negative predictive values for different levels of the two scores

Threshold of Linear predictor in model for patients eligible for escalation to ICU	Positive predictive value (% next day escalation to ICU or death among patients with a score greater than threshold)	Negative predictive value (% not having next day escalation to ICU or death among patients with a score lower than threshold)
0	1.4%	99.8%
1	6.5%	99.5%
2	13.5%	99.2%
3	19.8%	99.0%
4	31.8%	98.8%
5	42.6%	98.7%
6	46.2%	98.6%
Threshold of linear predictor in model for patients ineligible for escalation to ICU	Positive predictive value (% next day death among patients with a score greater than threshold)	Negative predictive value (% not having next day death among patients with a score lower than threshold)
0	2.9%	99.1%
1	15.2%	98.2%
2	26.1%	97.7%
3	46.6%	97.3%
4	62.2%	97.1%

Web Figure 1

Cohort from first SARS-CoV-2 date between 21 February 2020 until 30 June 2020

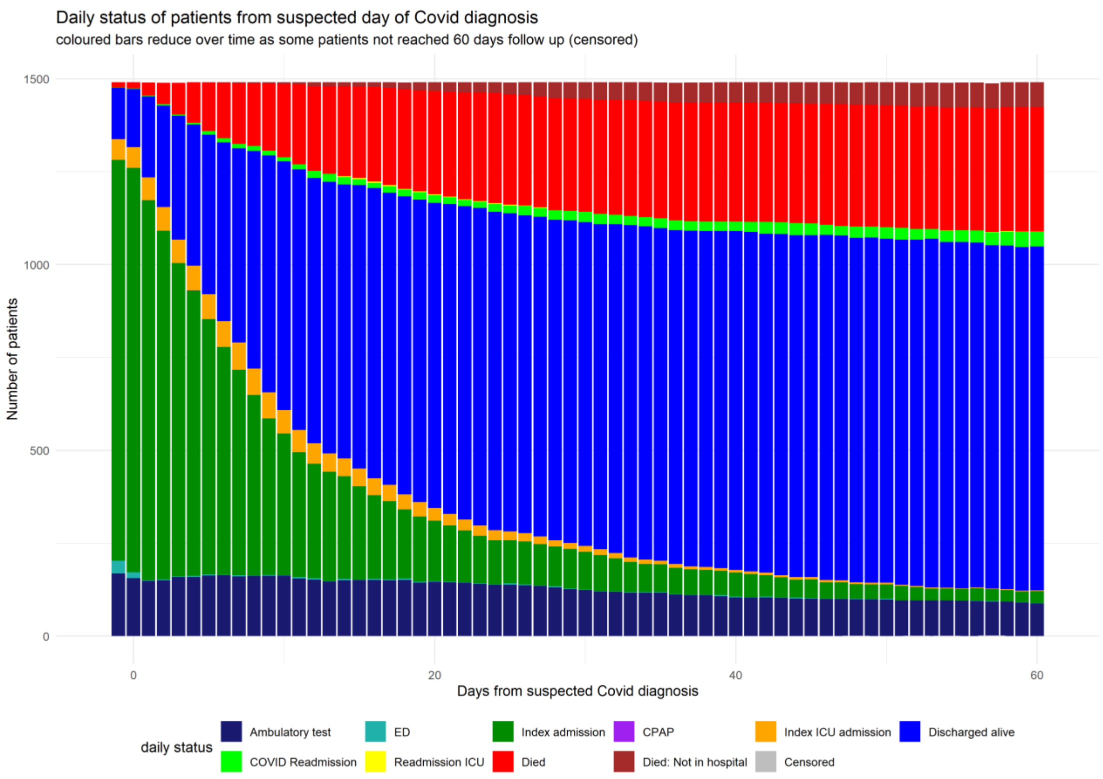

Web Figure 2A

Flow chart of all SARS-COV-2 patients in the derivation cohort until 30th June 2020

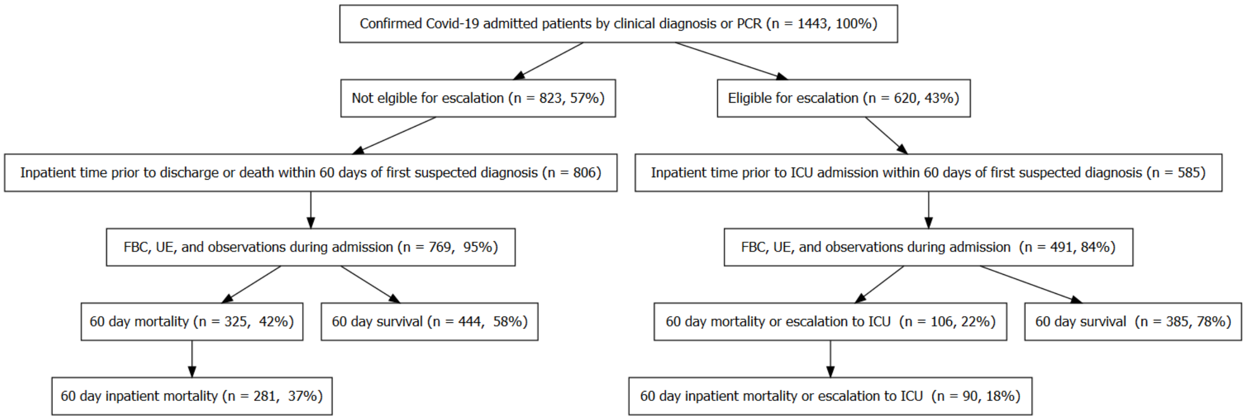

Web Figure 2B

Flow chart of all SARS-COV-2 patients in the validation cohort from 1st July 2020

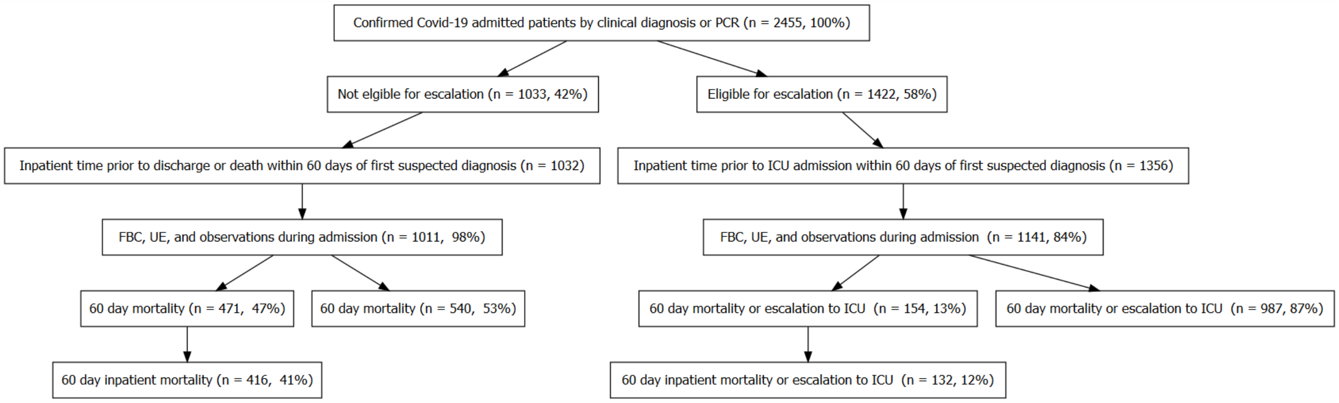

Web Figure 3

Longitudinal smoothed means of selected observations and blood tests by observed worst outcome

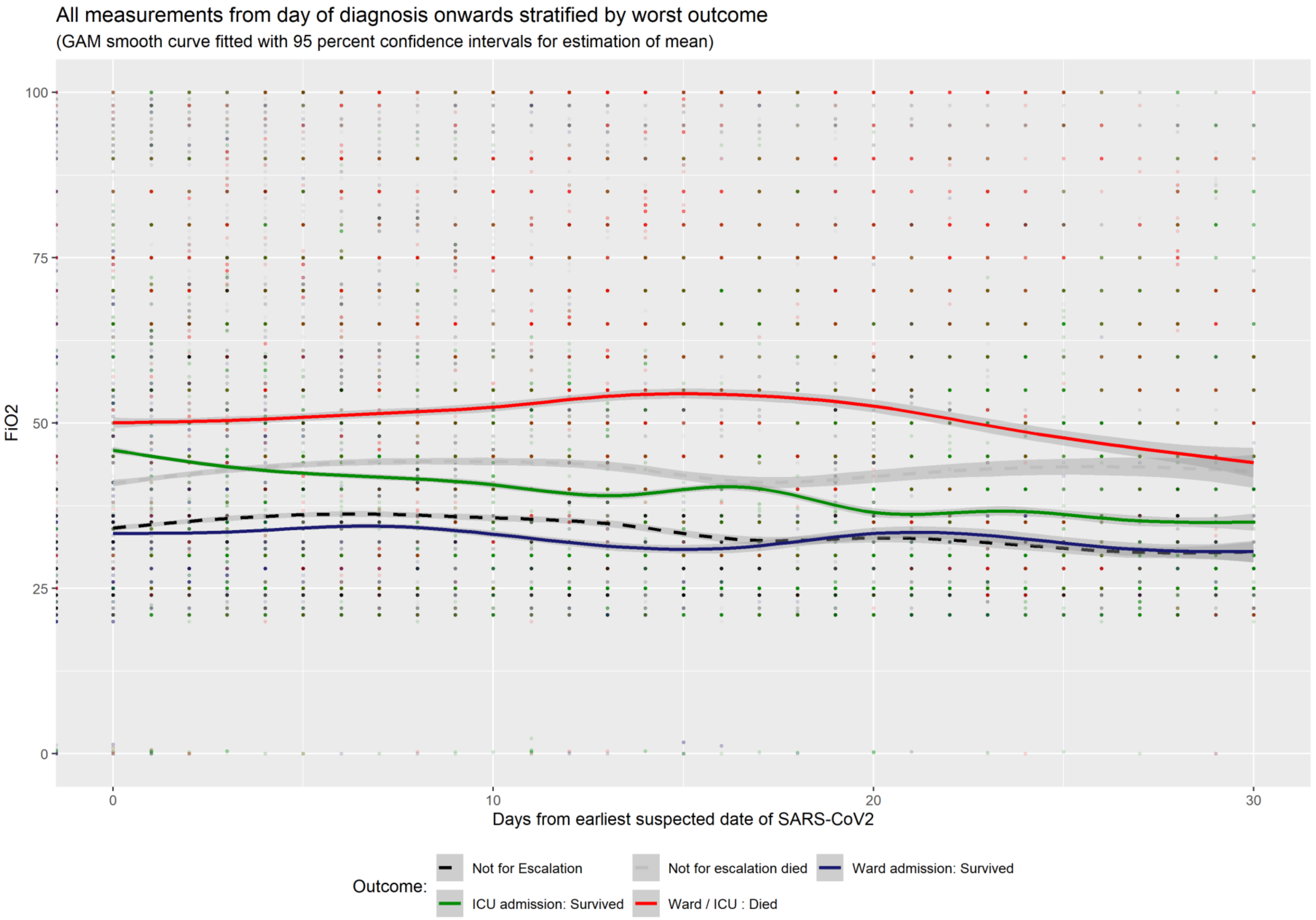

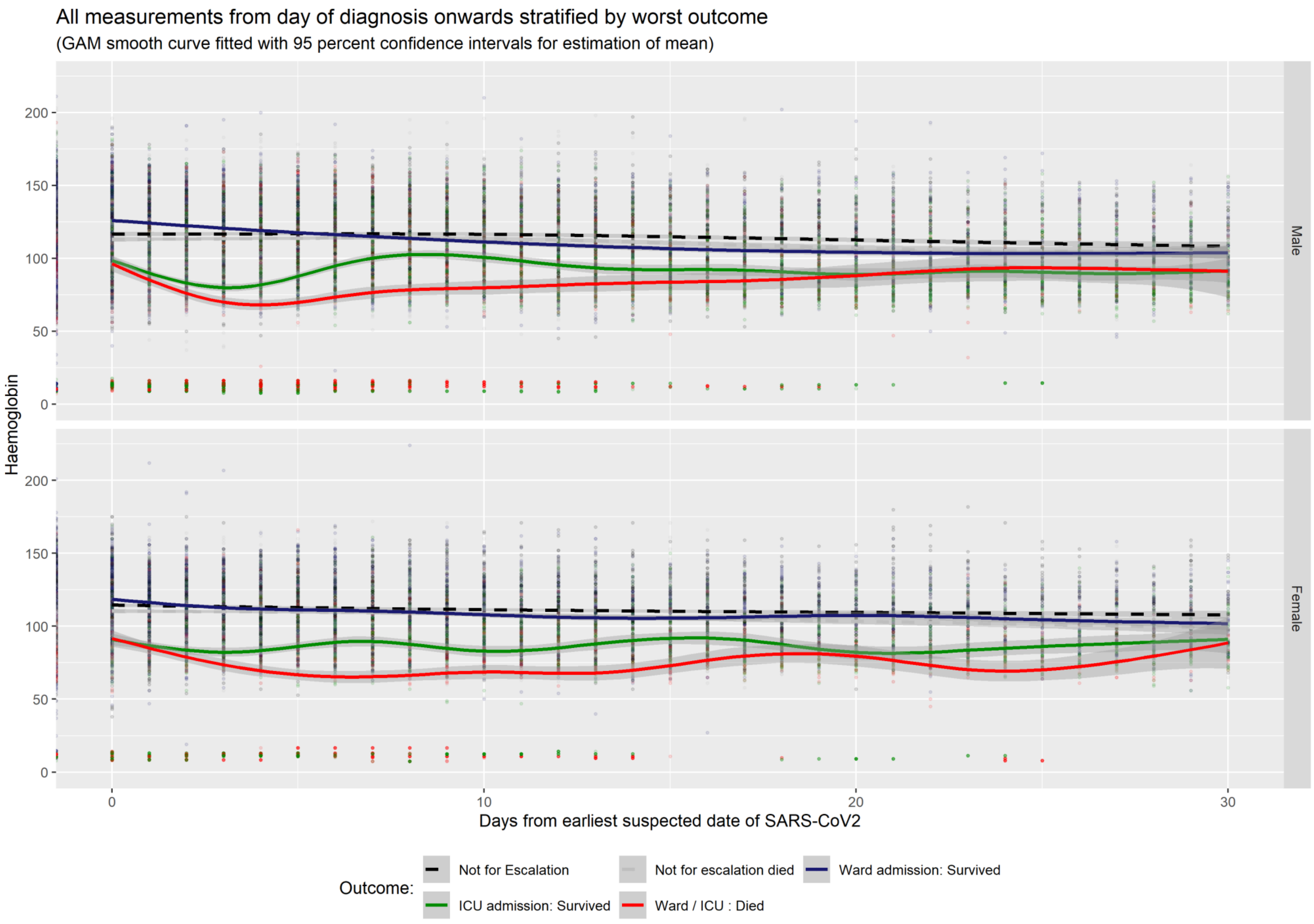
All measurements from day of diagnosis onwards stratified by worst outcome
(GAM smooth curve fitted with 95 percent confidence intervals for estimation of mean)
Haemoglobin
Male
Female
Days from earliest suspected date of SARS-CoV2
Outcome:
Not for Escalation
Not for escalation died
Ward admission: Survived
ICU admission: Survived
Ward / ICU : Died

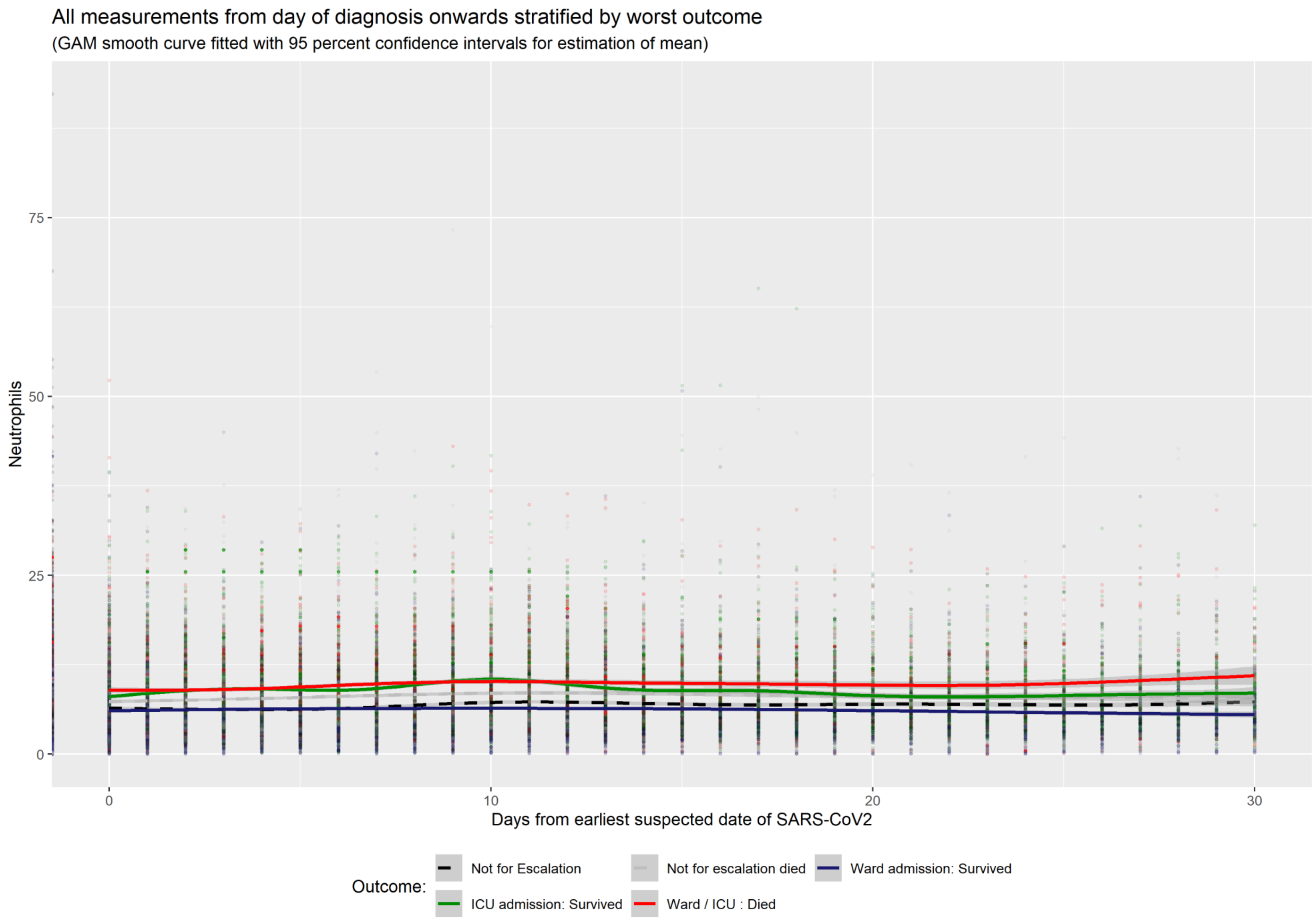
All measurements from day of diagnosis onwards stratified by worst outcome
(GAM smooth curve fitted with 95 percent confidence intervals for estimation of mean)
Neutrophils
0
25
50
75
0
10
20
30
Days from earliest suspected date of SARS-CoV2
Outcome:
Not for Escalation
Not for escalation died
Ward admission: Survived
ICU admission: Survived
Ward / ICU : Died

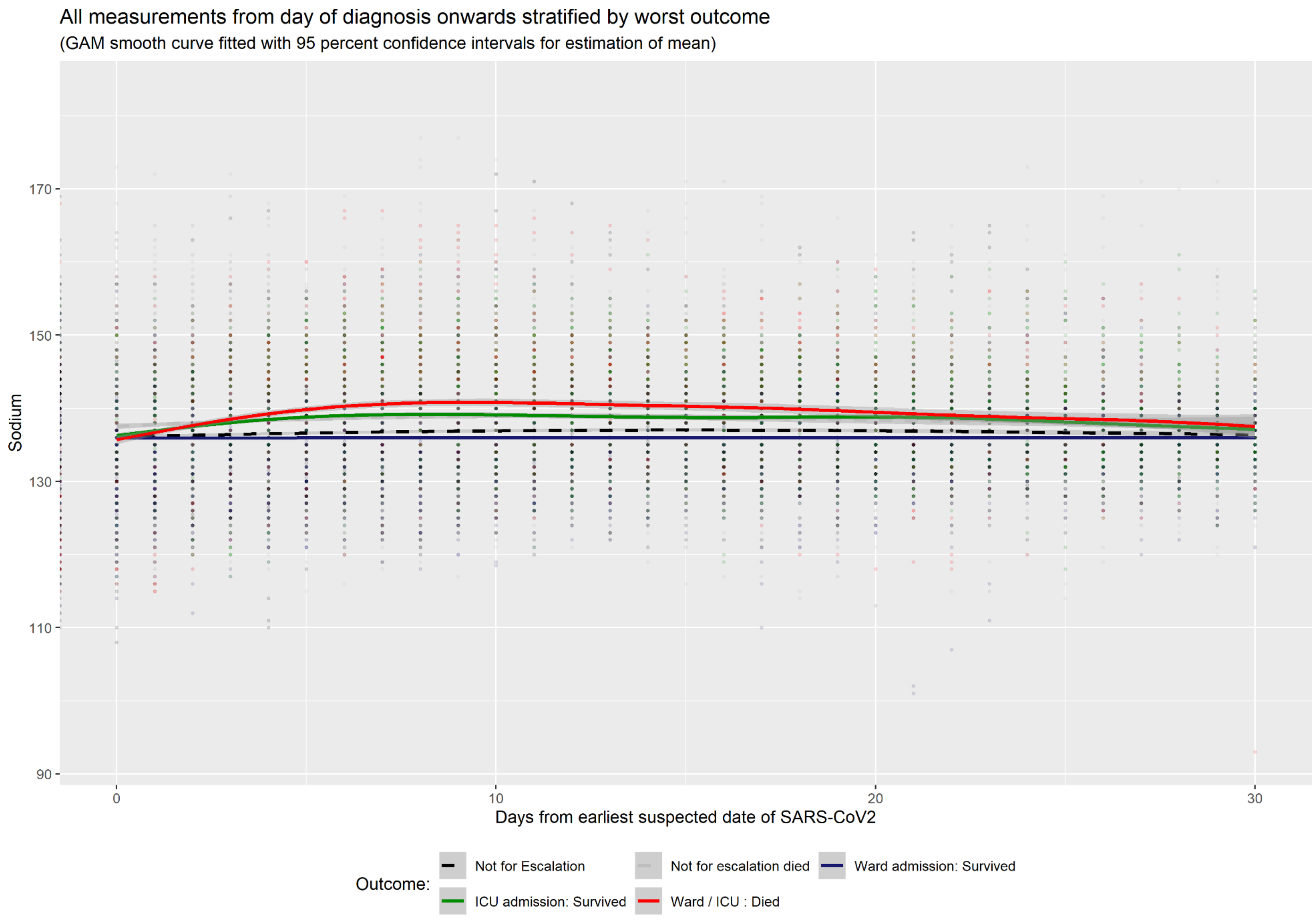
All measurements from day of diagnosis onwards stratified by worst outcome
(GAM smooth curve fitted with 95 percent confidence intervals for estimation of mean)
Sodium
170
150
130
110
90
0
10
20
30
Days from earliest suspected date of SARS-CoV2
Outcome:
Not for Escalation
Not for escalation died
Ward admission: Survived
ICU admission: Survived
Ward / ICU : Died

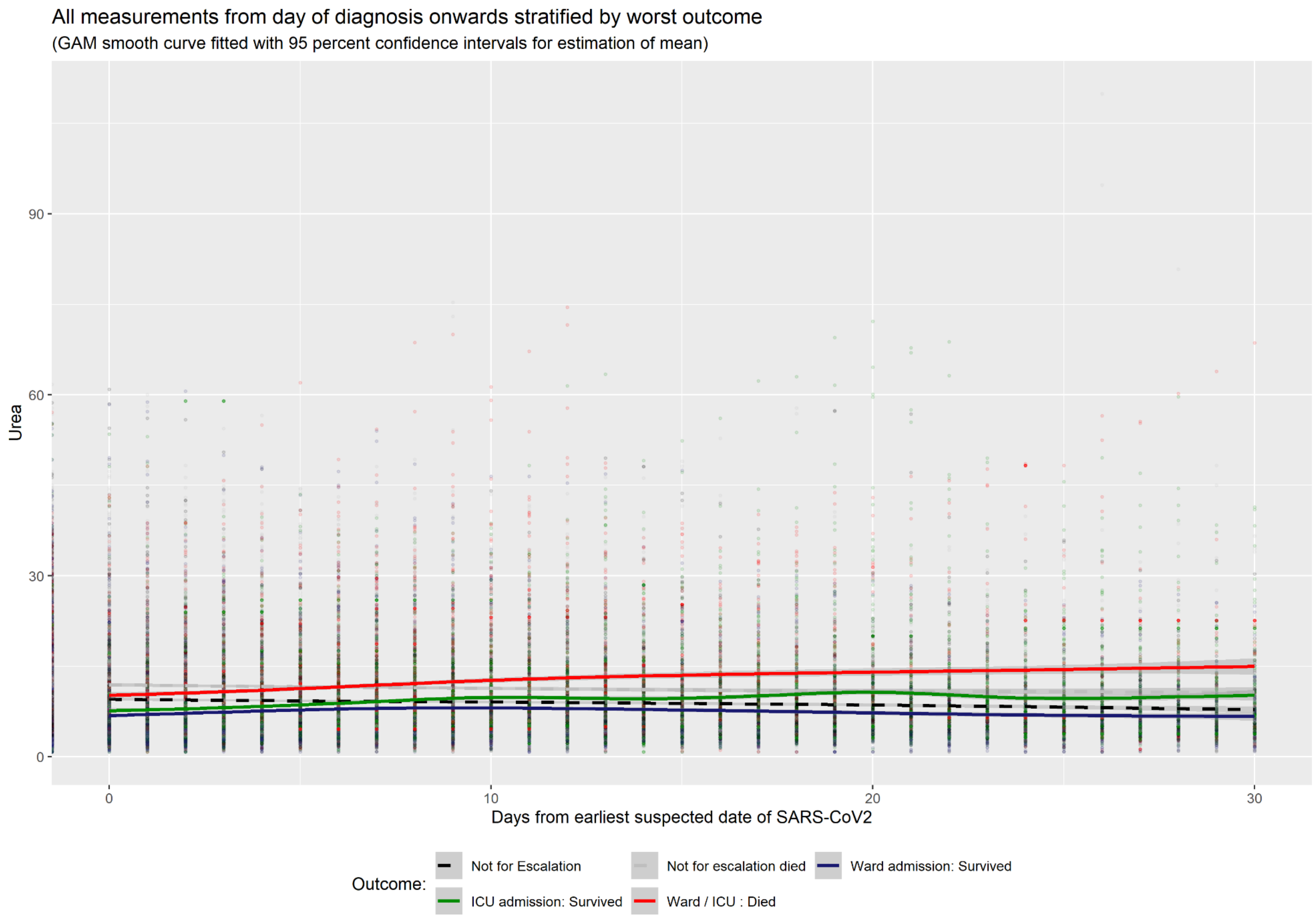
All measurements from day of diagnosis onwards stratified by worst outcome
(GAM smooth curve fitted with 95 percent confidence intervals for estimation of mean)
Urea
90
60
30
0
0
10
20
30
Days from earliest suspected date of SARS-CoV2
Outcome:
Not for Escalation
Not for escalation died
Ward admission: Survived
ICU admission: Survived
Ward / ICU : Died

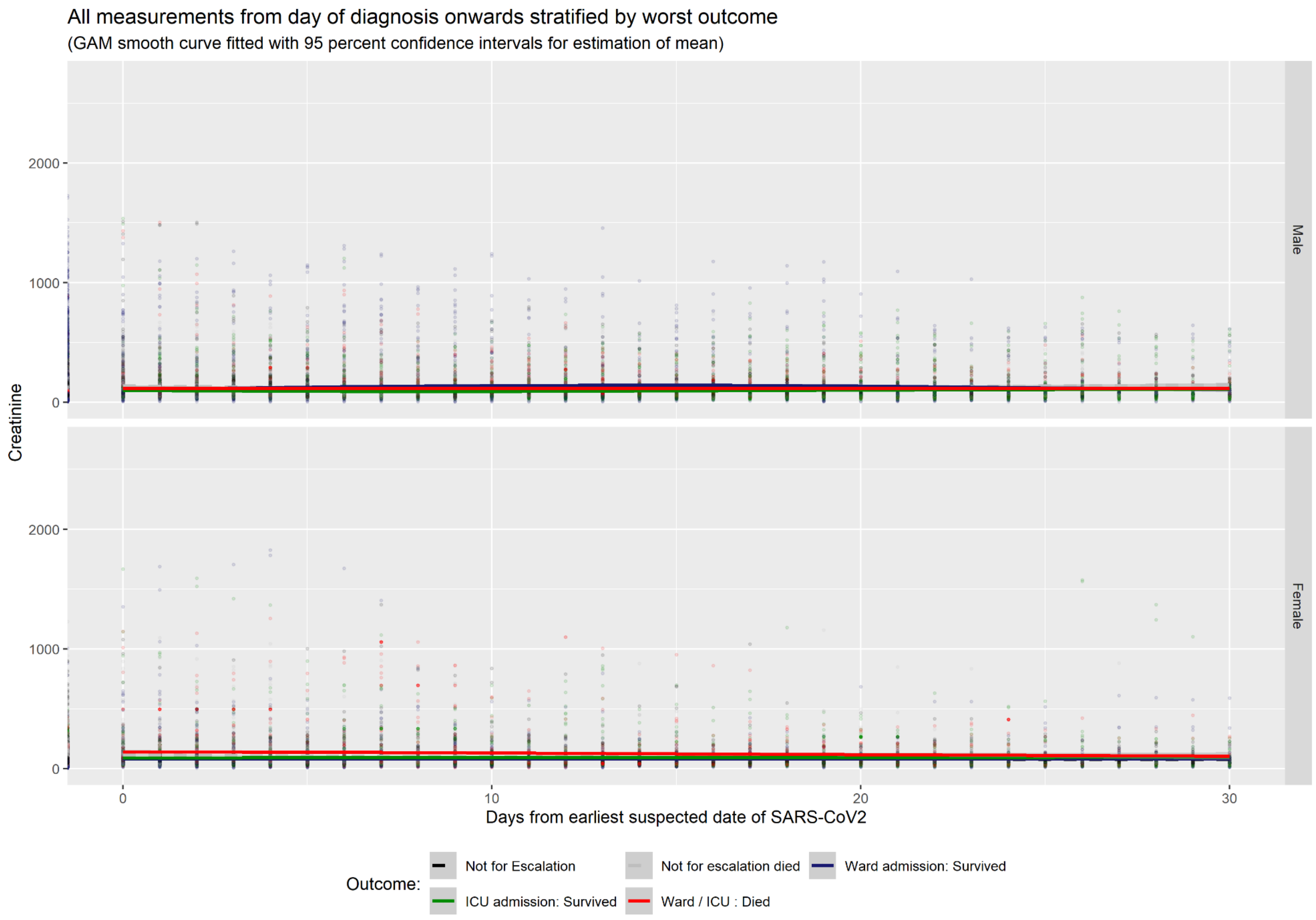
All measurements from day of diagnosis onwards stratified by worst outcome
(GAM smooth curve fitted with 95 percent confidence intervals for estimation of mean)
Creatinine
2000
1000
0
Male
Female
0
10
20
30
Days from earliest suspected date of SARS-CoV2
Outcome:
Not for Escalation
Not for escalation died
Ward admission: Survived
ICU admission: Survived
Ward / ICU : Died

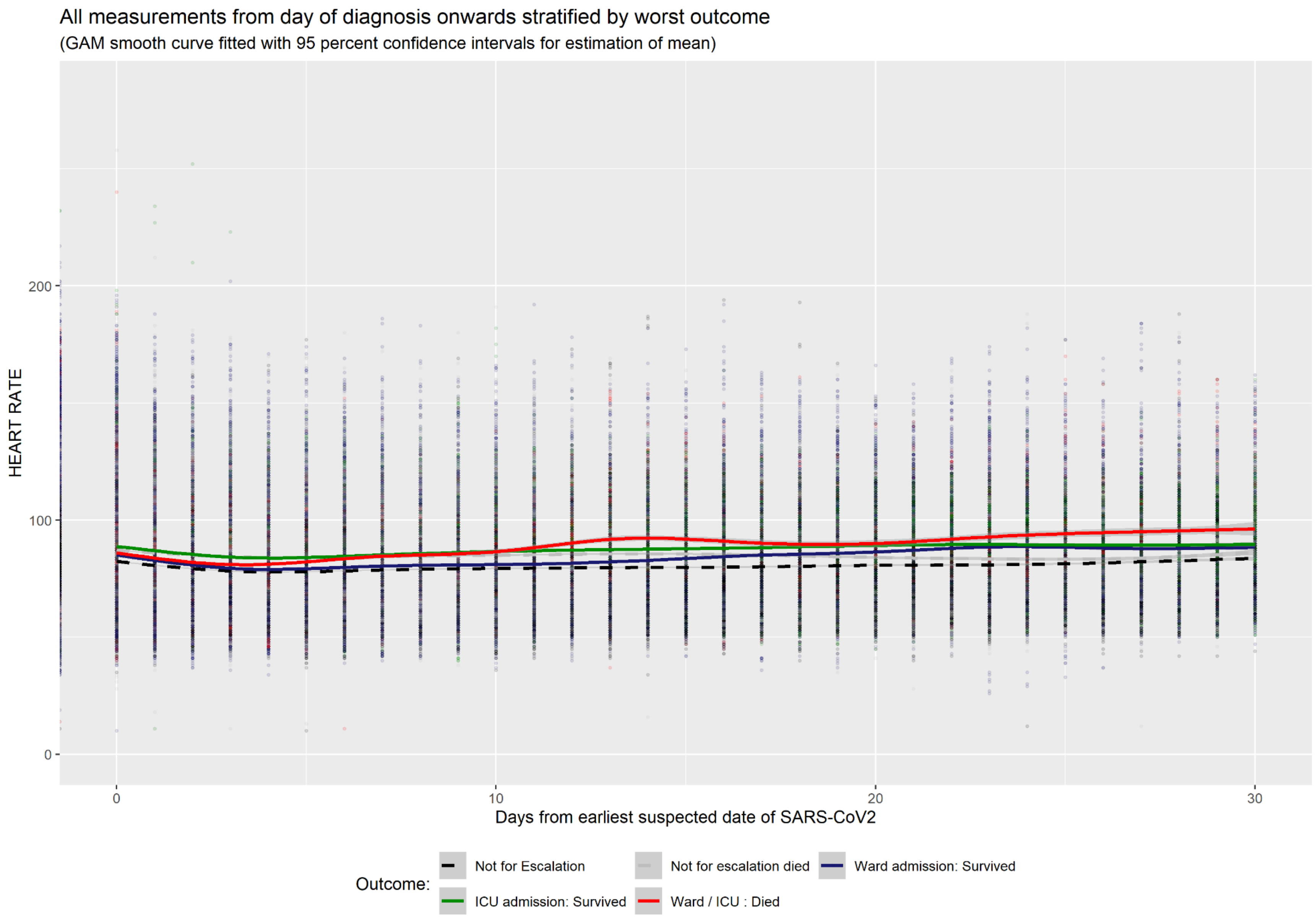
All measurements from day of diagnosis onwards stratified by worst outcome
(GAM smooth curve fitted with 95 percent confidence intervals for estimation of mean)
HEART RATE
200
100
0
0
10
20
30
Days from earliest suspected date of SARS-CoV2
Outcome:
Not for Escalation
Not for escalation died
Ward admission: Survived
ICU admission: Survived
Ward / ICU : Died

Web Figure 4

Schoenfeld residuals versus time for each covariate in the next day ICU escalation model

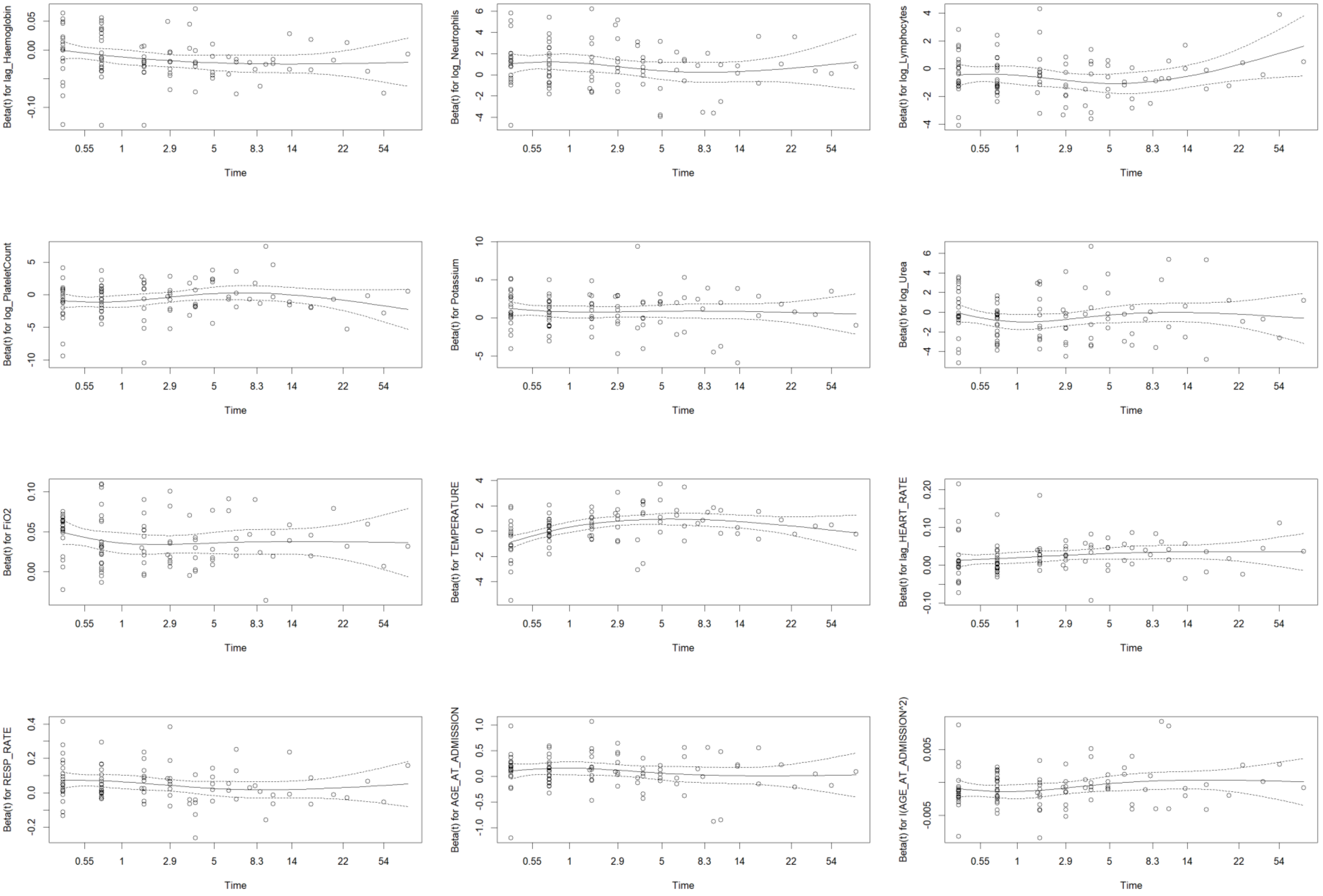

Web Figure 5

ROC curves and AUC for daily prediction of next day escalation from time varying model by day of disease course within patients eligible for escalation to ICU 1 July 2020 until 31 December 2020 in validation cohort

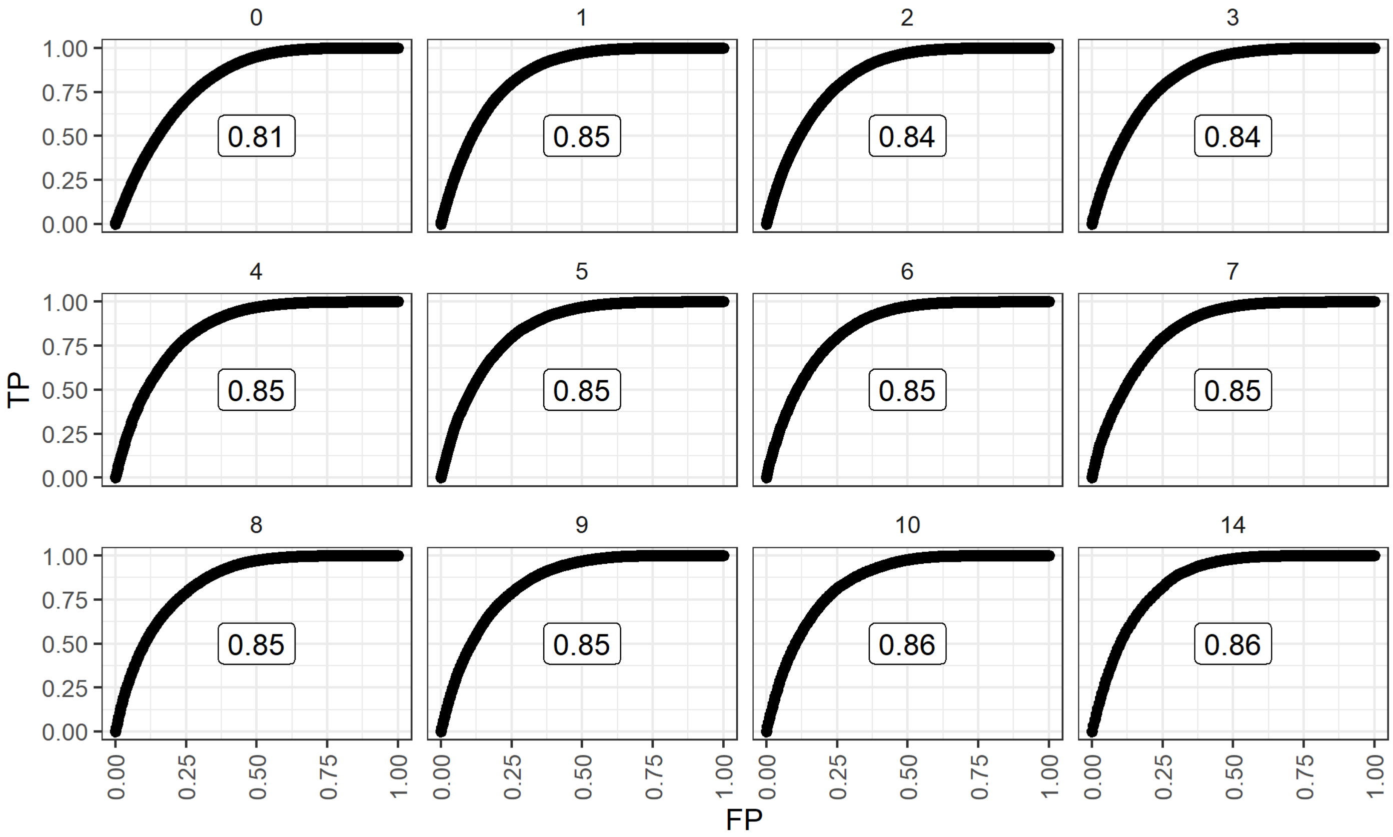

Web Figure 6

ROC curves and AUC for daily prediction of next day mortality by day of disease course for patients ineligible for escalation to ICU 1 July 2020 until 31 December 2020 in validation cohort

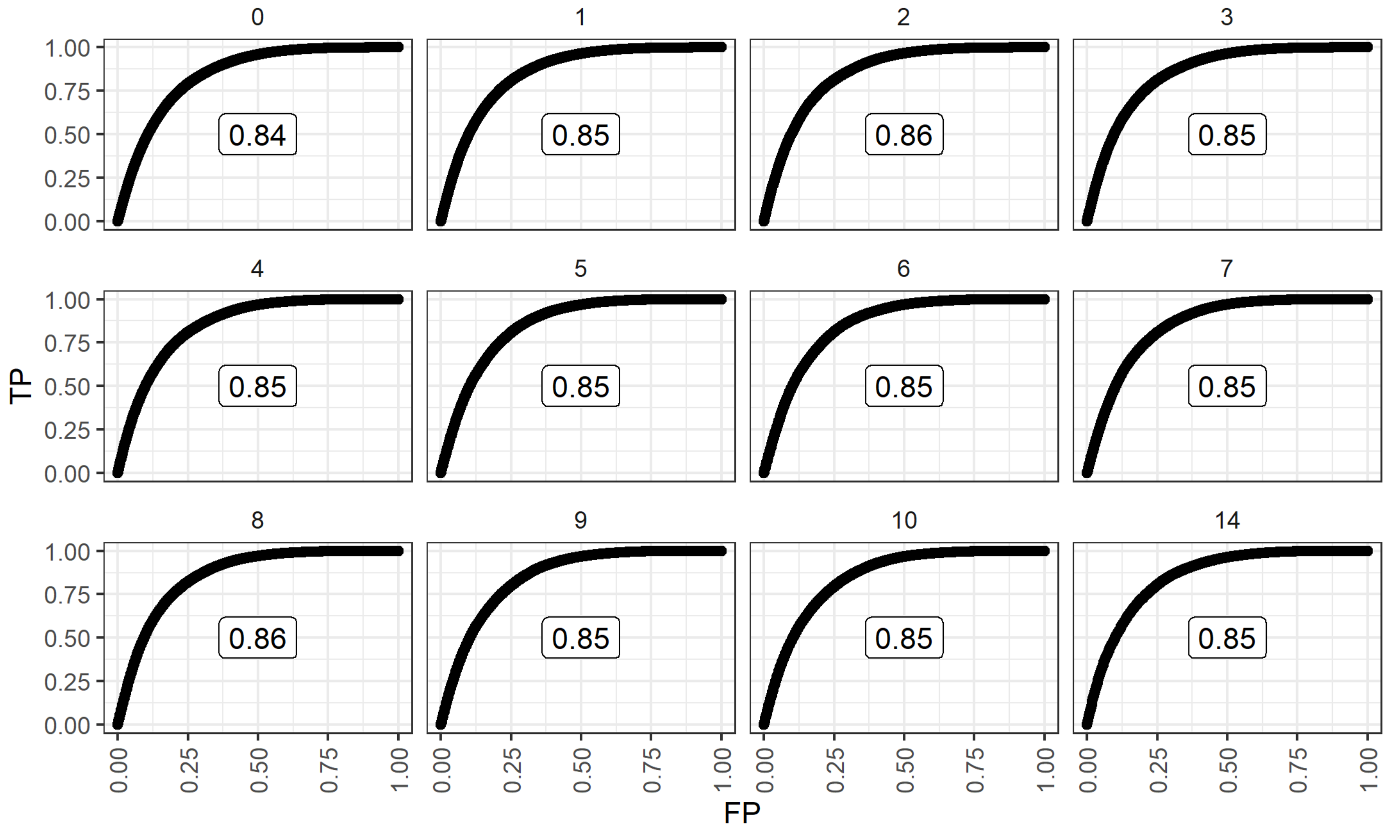

Web Figure 7

Baseline survival curves from fitted Cox proportional hazard models in table 3 in the derivation cohort, with 95% confidence intervals. Calculation set at mean values of cohort (model linear predictor calculations centered on mean values).

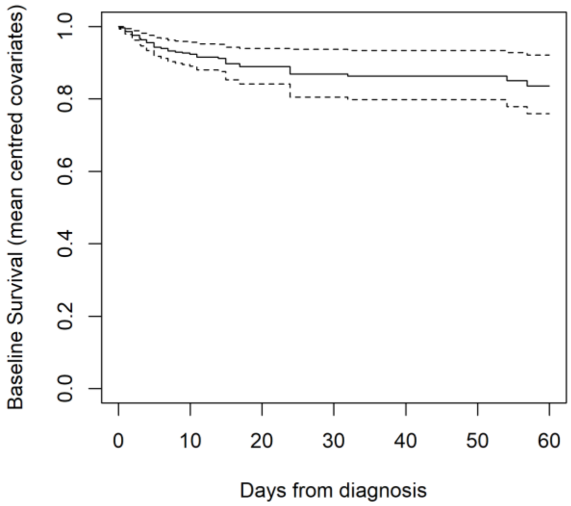	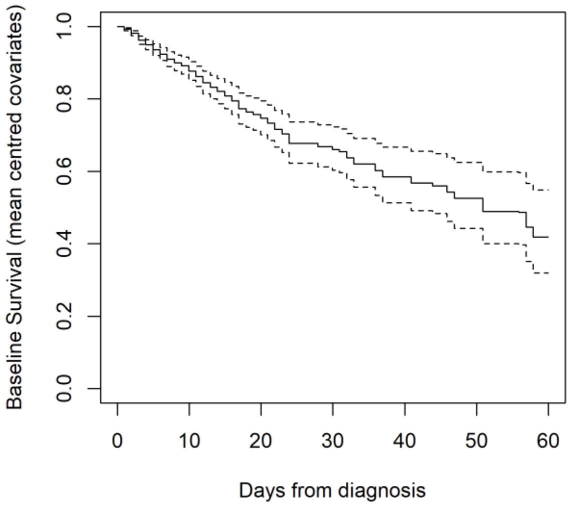
(A) Patients eligible for next-day escalation to ICU	(B) Patients ineligible for next-day escalation to ICU

Web Figure 8

Schoenfeld residuals versus time for each covariate in the next day mortality model

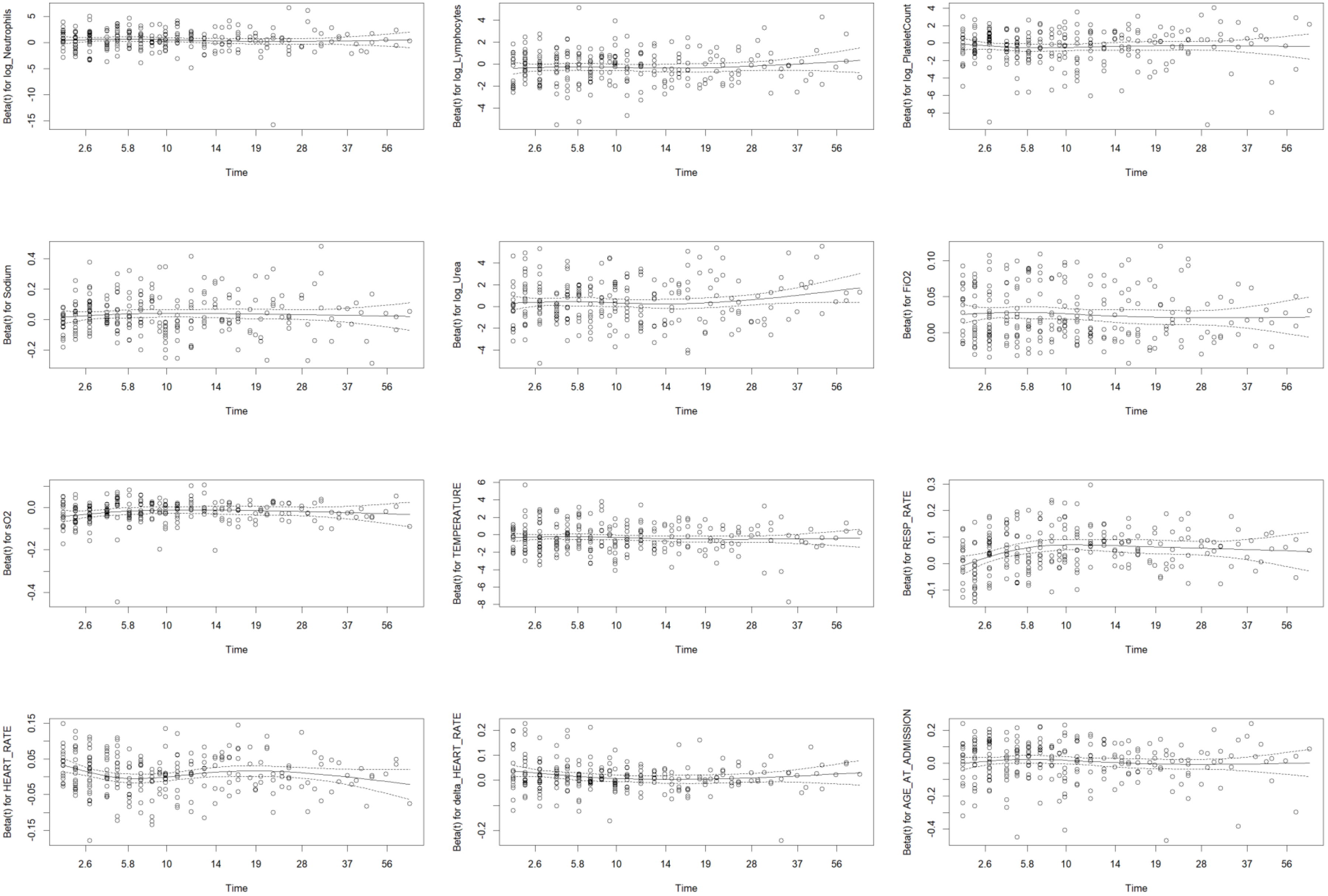

Web Figure 9

Calibration curve for next day prediction of ICU admission or death in the validation cohort for eligible patients for ICU, and for next day death for ineligible patients for ICU

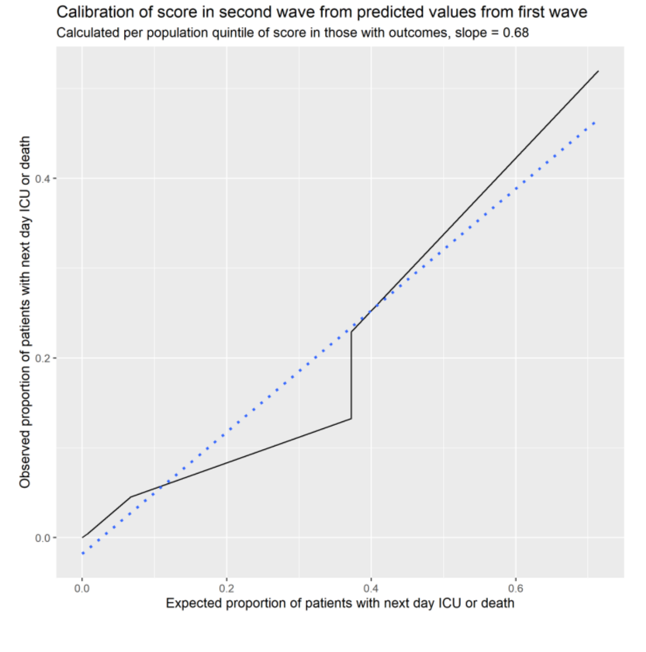

(A) Patients eligible for next day escalation to ICU

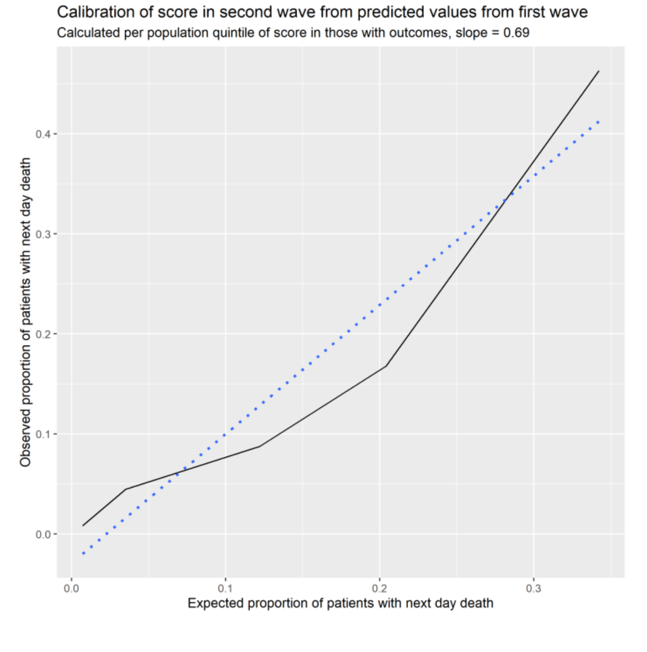

(B) Patients ineligible for next day escalation to ICU
