# Supplementary material for: Predicting need for escalation of care or death from repeated daily clinical observations and laboratory results in patients with SARS-CoV-2
Source: Am J Epidemiol. 2022 Jul 22:kwac126. doi: 10.1093/aje/kwac126 (PMC9384527; doi:10.1093/aje/kwac126)
Supplement: Supplementary file 1 [file web_material_kwac126.docx]

**Predicting need for escalation of care or death from repeated daily clinical observations and laboratory results in patients with SARS-CoV-2**

Colin J Crooks, Joe West, Andrew Fogarty, Joanne R Morling, Matthew J Grainge, Sherif Gonem, Mark Simmonds, Andrea Race, Irene Juurlink, Steve Briggs, Simon Cruickshank, Susan Hammond-Pears and Timothy R Card

**Web material contents:**

Web Table 1	2

Web Table 2	3

Web Table 3	4

Web Table 4	5

Web Table 5	6

Web Table 6	7

Web Figure 1	8

Web Figure 2a	9

Web Figure 2b	9

Web Figure 3	10

Web Figure 4	17

Web Figure 5	18

Web Figure 6:	19

Web Figure 7	20

Web Figure 8	21

Web Figure 9	22

## Web Table 1 

Range of sample size calculations performed

|  |  | Calculated using pmsampsize command in Stata (Riley, RD, Snell, KIE, Ensor, J, et al. Minimum sample size for developing a multivariable prediction model: PART II ‐ binary and time‐to‐event outcomes. *Statistics in Medicine*. 2019; 38: 1276– 1296. [https://doi.org/10.1002/sim.7992](https://doi.org/10.1002/sim.7992)) |  |  |
| --- | --- | --- | --- | --- |
|  |  | Minimum sample size to meet requirements with 10 parameters in model and model r2 = 0.15 |  |  |
| Prevalence | Expected number of events | Global shrinkage (0.9) | Difference between r2 and adjusted r2 <0.05 | Precision of average outcome risk +/-0.05 |
| 0.12 | 66 | 549 | 349 | 163 |
| 0.15 | 83 | 549 | 318 | 198 |
| 0.18 | 99 | 549 | 298 | 227 |
| 0.21 | 116 | 549 | 283 | 255 |
| 0.24 | 132 | 549 | 270 | 281 |
| 0.27 | 149 | 549 | 263 | 303 |
|  |  |  |  |  |
|  |  |  |  |  |
| Prevalence |  | Global shrinkage (0.89) | Difference between r2 and adjusted r2 <0.05 | Precision of average outcome risk +/-0.05 |
| 0.12 | 60 | 493 | 349 | 163 |
| 0.15 | 74 | 493 | 318 | 198 |
| 0.18 | 89 | 493 | 298 | 227 |
| 0.21 | 104 | 493 | 283 | 255 |
| 0.24 | 119 | 493 | 270 | 281 |
| 0.27 | 134 | 493 | 263 | 303 |

## Web Table 2

Sociodemographic and other characteristics on admission of the cohort who were admitted to hospital with SARS-COV-2 21 February 2020 until 30 June 2020

|  | Admission: Clinical diagnosis only | Admission: PCR positive |
| --- | --- | --- |
| N | 403 | 1040 |
| Age (years) median (IQR) | 77 (62, 85) | 76 (60, 84) |
| Male | 198 (49%) | 553 (53%) |
| Other or not stated ethnic group | 58 (14%) | 197 (19%) |
| Black/Mixed ethnic group | 10 (2%) | 45 (4%) |
| Indian/Pakistani ethnic group | 9 (2%) | 47 (5%) |
| White ethnic group | 326 (81%) | 751 (72%) |
| 30 day mortality | 83 (21%) | 282 (27%) |
| Died out of hospital | 13 (3%) | 28 (3%) |
| 30 day ICU admission | 21 (5%) | 130 (12%) |
| Length of stay, days (IQR) | 8 (4, 13) | 8 (3, 18) |
| For escalation/CPR | 153 (38%) | 467 (45%) |
| NEWS2, median (IQR) | 3 (2, 5) | 3 (2, 5) |
| ISARIC-4C, median (IQR) | 10 (7, 12) | 10 (6, 12) |
| BMI <20, n, % | 75 (19%) | 184 (18%) |
| BMI >30, n, % | 93 (23%) | 289 (28%) |
| Smoking, n, % | 73 (18%) | 87 (8%) |
| Vaping, n, % | 27 (7%) | 40 (4%) |
| Alcohol risk, n, % | 57 (14%) | 145 (14%) |
| Charlson Index, median (IQR) | 2 (1, 4) | 2 (0, 3) |

## Web Table 3

Test for slope of zero for Schoenfeld residuals versus time for next day ICU escalation model

| Model of risk of next day escalation to ICU | Test for slope of zero for Schoenfeld residuals versus time: |
| --- | --- |
|  |  |
|  | p value (Chi2) |
| Lagged change in daily mean Haemoglobin | 0.02 |
| log(Neutrophil count) | 0.94 |
| log(Lymphocyte count) | 0.66 |
| Log(Daily mean platelet count) | 0.83 |
| Daily mean Potassium | 0.83 |
| Lagged change in daily mean Urea | 0.53 |
| FiO2 (%) | 0.20 |
| Highest Temperature (^o^C) | 0.0003 |
| Lagged change in daily highest heart rate | 0.21 |
| Highest daily respiratory rate | 0.22 |

## Web Table 4

Calculation of linear predictors for models used for validation in second wave

Linear predictor for next day escalation/death model for patients eligible for escalation =

-0.0125674 * (lag_Haemoglobin - 9.213636) + 

0.8701899 * (log_Neutrophils - 1.537172) +   

-0.5621189 * (log_Lymphocytes - 0.083262) +  

-0.6348783 * (log_PlateletCount - 5.484431) + 						

0.9202828 * (Potassium - 4.132611) +

-0.4942963 * (log_Urea - 1.731216) +		

0.2340765 * (TEMPERATURE - 37.189060) +		

0.0531105 * (RESP_RATE - 21.033282) +		

0.0225751 * (lag_HEART_RATE - 6.655470) +		

0.0398151 * (FiO2 - 28.850724) +    

0.1032351 * (AGE_AT_ADMISSION - 57.969800) +

-0.0007869 * (AGE_AT_ADMISSION^2 - 3824.085054)

Linear predictor for next day death model for patients ineligible for escalation =

0.4574248 * (log_Neutrophils - 1.6873937) +

-0.2822816 * (log_Lymphocytes - -0.0250936) +

-0.3771835 * (log_PlateletCount - 5.4290112) +

0.0330612 * (Sodium - 137.3990841) +

0.4446858 * (log_Urea - 2.0373875) +

-0.3360895 * (TEMPERATURE - 36.99199) +

0.0494824 * (RESP_RATE - 20.8943202) +

0.0198026 * (HEART_RATE - 90.2390630)   +

0.0164881 * (delta_HEART_RATE - -0.1344529)   +

0.0251410 * (FiO2 - 30.3385603) +

-0.0215520 * (sO2 - 93.3695048) +

0.0094745 * (AGE_AT_ADMISSION - 79.9594609)

## Web Table 5 

Test for slope of zero for Schoenfeld residuals versus time for next day mortality model

|  |  |
| --- | --- |
| Model of risk of next day mortality | Test for slope of zero for Schoenfeld residuals versus time: |
|  |  |
|  | p value (Chi2) |
| log(Daily mean Neutrophil count) | 0.65 |
| log(Daily mean Lymphocyte count) | 0.48 |
| log(Daily mean Platelet count) | 0.86 |
| Daily mean Sodium | 0.25 |
| log(Daily mean Urea) | 0.21 |
| Highest FiO2 (%) | 0.21 |
| Lowest daily Oxygen saturations (%) | 0.07 |
| Daily highest Temperature | 0.19 |
| Daily highest respiratory rate | 0.12 |
| Daily highest heart rate | 0.20 |
| Within day change in heart rate | 0.04 |

## Web Table 6

Positive and negative predictive values for different levels of the two scores

| Threshold of Linear predictor in model for patients eligible for escalation to ICU | Positive predictive value (% next day escalation to ICU or death among patients with a score greater than threshold) | Negative predictive value (% not having next day escalation to ICU or death among patients with a score lower than threshold) |
| --- | --- | --- |
| 0 | 1.4% | 99.8% |
| 1 | 6.5% | 99.5% |
| 2 | 13.5% | 99.2% |
| 3 | 19.8% | 99.0% |
| 4 | 31.8% | 98.8% |
| 5 | 42.6% | 98.7% |
| 6 | 46.2% | 98.6% |
|  |  |  |
| Threshold of linear predictor in model for patients ineligible for escalation to ICU | Positive predictive value (% next day death among patients with a score greater than threshold) | Negative predictive value (% not having next day death among patients with a score lower than threshold) |
| 0 | 2.9% | 99.1% |
| 1 | 15.2% | 98.2% |
| 2 | 26.1% | 97.7% |
| 3 | 46.6% | 97.3% |
| 4 | 62.2% | 97.1% |

## Web Figure 1 

Cohort from first SARS-CoV-2 date between 21 February 2020 until 30 June 2020

## Web Figure 2a 

Flow chart of all SARS-COV-2 patients in the derivation cohort until 30th June 2020

## **Web Figure 2b** 

Flow chart of all SARS-COV-2 patients in the validation cohort from 1st July 2020

## Web Figure 3 

Longitudinal smoothed means of selected observations and blood tests by observed worst outcome

## Web Figure 4 

Schoenfeld residuals versus time for each covariate in the next day ICU escalation model

## Web Figure 5  

ROC curves and AUC for daily prediction of next day escalation from time varying model by day of disease course within patients eligible for escalation to ICU 1 July 2020 until 31 December 2020 in validation cohort

| Web Figure 6: |  |
| --- | --- |
| ROC curves and AUC for daily prediction of next day mortality by day of disease course for patients ineligible for escalation to ICU 1 July 2020 until 31 December 2020 in validation cohort |  |

## Web Figure 7

Baseline survival curves from fitted Cox proportional hazard models in table 3 in the derivation cohort, with 95% confidence intervals. Calculation set at mean values of cohort (model linear predictor calculations centred on mean values).

|  |  |
| --- | --- |
| (a) Patients eligible for next day escalation to ICU | (b) Patients ineligible for next day escalation to ICU |

## Web Figure 8 

Schoenfeld residuals versus time for each covariate in the next day mortality model

## Web Figure 9

Calibration curve for next day prediction of ICU admission or death in the validation cohort for eligible patients for ICU, and for next day death for ineligible patients for ICU

|  |  |
| --- | --- |
| (a) Patients eligible for next day escalation to ICU | (b) Patients ineligible for next day escalation to ICU |
